# PlantGFM: A Genomic Foundation Model for Discovery and Creation of Plant Genes

**DOI:** 10.1002/advs.75772

**Published:** 2026-05-20

**Authors:** Changhao Li, Qizhe Zhang, Hanchen Chen, Kepeng Lin, Chengfang Luo, Mengying Yang, Wei Xu, Fan Yao, Jianbing Yan, Qing‐Yong Yang, Xuehai Hu

**Affiliations:** ^1^ Hubei Hongshan Laboratory Wuhan China; ^2^ College of Informatics Agricultural Bioinformatics Key Laboratory of Hubei Province Huazhong Agricultural University Wuhan China; ^3^ Yazhouwan National Laboratory Sanya China; ^4^ School of Electronic Information and Communications Huazhong University of Science and Technology Wuhan China; ^5^ College of Biomedicine and Health Huazhong Agricultural University Wuhan Hubei China; ^6^ College of Life Science and Technology Huazhong Agricultural University Wuhan Hubei China; ^7^ National Key Laboratory of Crop Genetic Improvement Huazhong Agricultural University Wuhan China

**Keywords:** AI‐HK fusion, de novo gene design, genomic foundation model, hyena architecture, plant synthetic biology

## Abstract

The artificial intelligence (AI)‐driven generation of genetic sequences holds transformative potential for addressing global challenges in agriculture, medicine, and bioenergy. Traditional approaches including hybridization, mutagenesis, and CRISPR‐based editing enable targeted modification of endogenous DNA, yet remain constrained by natural sequence diversity. We here introduce PlantGFM, an application of the Hyena operator within a plant‐oriented genomic foundation model, which was pre‐trained on 10.84 billion nucleotides from 12 plant species and supports long‐context (64 kb) prediction and sequence generation within a unified architecture. After fine‐tuning on 10 annotated plant genomes, PlantGFM matched or exceeded the performance of specialized gene prediction tools. Beyond reproducing natural genes, it enables de novo design of novel candidates through the emergence capability of AI. Seven candidates selected through an AI–Human Knowledge fusion screening pipeline all showed transcriptional activity in Nicotiana benthamiana, two with stable protein expression—representing the first demonstration of DNA–RNA–protein expression of Large Language Model‐generated sequences in plants. As a proof of concept, PlantGFM also exhibits emergent abilities in generating plant NLR genes. Our findings establish the feasibility of LLM technology for de novo plant gene design, providing a foundation for plant synthetic biology and AI‐assisted breeding.

## Graphical abstract

A plant genomic foundation model pre‐trained on 12 species enables both accurate gene prediction and de novo gene design. Through AI‐human knowledge screening, seven designed sequences showed transcriptional activity in plants, with two expressing stable proteins—demonstrating the first DNA‐RNA‐protein expression of LLM‐generated genes in plants and establishing feasibility for AI‐driven synthetic biology.

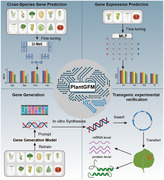

## 1 Introduction

The capacity to read, write, and engineer genetic sequences underpins transformative advances in modern biotechnology, offering powerful tools for improving crop resilience, precision medicine, and sustainable bioengineering [1, 2, 3, 4]. Classical techniques—including hybridization breeding [5], random mutagenesis [6], and CRISPR‐based editing [7]—have revolutionized the manipulation of endogenous DNA sequences, enabling precise yet bounded modifications within natural genomic constraints. However, these methods are inherently limited in their capacity to design and construct novel genetic architectures that go beyond naturally occurring variants.

*De novo* gene synthesis and synthetic biology are expected to offer a new avenue for addressing the challenges of rationally designing artificial sequences with customized functions [8], from climate‐resilient crops to bioengineered microbial factories [3, 4, 9]. Despite the widespread adoption of CRISPR technologies, large‐scale *de novo* gene design remains a formidable challenge, primarily due to the complexity of gene structure and the lack of computational tools capable of modeling long, biologically valid sequences. In this nascent field, the earliest breakthroughs have been achieved in the *de novo* design of regulatory elements such as promoters, enhancers, and 5' UTRs [10, 11, 12, 13, 14, 15]. Among these efforts, a seminal study by Wang et al. used a Generative Adversarial Network (GAN) to *de novo* generate *E. coli* promoters, and in vivo validation confirmed that the generated sequences possessed promoter functionality [14]. Similarly, DeepCROSS employed an Adversarial Autoencoder (AAE) architecture trained on 1.8 million regulatory sequences from diverse bacterial genomes. The system combines a predictor model with a genetic algorithm to design species‐specific regulatory sequences for *E. coli* and *P. aeruginosa* [15]. Overall, most existing studies adopt generative models from the deep learning era, such as GANs, Variational AutoEncoder (VAE), and AAE, and the generated sequences are relatively short, usually limited to between 50 and 1,000 bp. However, those methods and models mentioned above cannot be directly transferred into the area of gene generation, because gene sequences involve much longer contexts (often several kb long) and are influenced by various factors, including the discrete exon‐intron structure, and the complex untranslated regions, making gene generation and design still challenging. Another thing that cannot be ignored is: despite the increasing number of studies on *de novo* regulatory sequence design, fewer than 1% of the generated sequences have been experimentally validated [10, 11, 12, 13, 14, 15].

Recently, with the great breakthroughs of Large Language Model (LLM) technology, the modeling of longer contexts has become feasible. DNABERT [16], DNABERT2 [17] and Nucleotide Transformer (NT) [18] represent some of the first pioneering applications built on the BERT architecture and have achieved success in modeling genomic data. However, their lack of sequence generation capability limits their use in synthetic biology and sequence design. Similarly, advanced architectures have revolutionized predictive modeling: ANNEVO [19] integrated Transformer [20] and Mixture of Experts (MoE) [21] techniques for ab initio gene annotation, while the recently introduced AlphaGenome [22] pushed the context window to an unprecedented 1 Mb to decode regulatory syntax and variant effects at single‐base resolution. Even so, despite setting new state‐of‐the‐art benchmarks in interpretation, both models primarily focus on structure prediction or functional inference rather than the generative design of novel sequences. If the focus is shifted toward genomic language models (GFMs) that are capable of performing generative tasks, megaDNA [23] can generate phage genomes with lengths ranging from 1 to 96 kb, while Generator [24] can generate coding DNA sequences for the histone and cytochrome P450 families, with each sequence within 2 kb. However, neither of the above two works have been experimentally validated, so their biological relevance remains uncertain. A recent breakthrough is Evo [25], a long‐context GFM capable of learning contexts up to 131 kb, which has learned the genomic language of prokaryotes and phages and has demonstrated the ability to generate entirely new CRISPR systems and transposons, showcasing its potential in ultra‐long sequence generation. Beyond model innovation, Evo 1.5 [26] introduces a DNA‐language–prompt–based gene generation paradigm. Unlike Evo and existing GFMs that rely on natural‐language prompts, Evo 1.5 uses DNA sequences themselves as prompts and demonstrates strong performance in toxin–antitoxin sequence generation tasks.

Although Evo and Evo 1.5 have demonstrated strong performance in prokaryotic systems, its training data are predominantly derived from prokaryotic genomes. Therefore, its ability to capture plant‐specific genomic features—such as complex genome architecture, large‐fragment transposon insertion and diverse structural variation—remains to be clarified. While plant‐oriented models such as AgroNT [27] and PDLLM [28] have attempted to address this gap by learning the genomic language of plant species, their relatively short context length (6 kb in AgroNT, and 2 kb in PDLLM) limits their capacity to model long genomic sequences. Although Evo 2 [29], which has not yet been officially published, incorporates species from across the entire tree of life as training samples, its performance on various plant genomic tasks remains unknown. A notable example is the maize domestication gene teosinte branched1 (*tb1*) [30], whose expression is regulated by a distant 60 kb upstream enhancer incorporating transposable element insertions. Furthermore, the gap between computational design and experimental validation hinders the translation of synthetic genes into real‐world applications, with only a few synthetic sequences validated in prokaryotic systems like bacteria, and plant systems still relatively blank.

Today, we have a new perspective to re‐examine plant genomics: viewing the genome as a kind of “language of life”, and AI provides a possible way to meet the goal of genomics by both deciphering existing genomic language and generating new language. We here introduce PlantGFM (Plant Genomic Foundation Model), an application of the Hyena operator within a plant‐oriented GFM trained on 10.84 billion nucleotides from 12 plant genomes, to contribute two key innovations to plant genomics. First, PlantGFM is capable of modeling genomic sequences up to 64 kb with single‐nucleotide resolution and supports both long‐context prediction and sequence generation within a unified architecture, enabling it to capture long‐range regulatory interactions essential for interpreting both coding and non‐coding genomic elements. Second, empowered by its innovation, PlantGFM leverages the strong emergent capabilities of AI to elegantly design entirely new candidate plant genes de novo. Benefiting from these innovations, PlantGFM achieves two major advances in plant genomics: Precision Gene Prediction, where it demonstrates competitive performance in cross‐species gene annotation of gene structures, including genes, exons, and coding sequences (CDS), compared to state‐of‐the‐art tools such as Helixer [31], ANNEVO [19], AUGUSTUS [32] and SegmentNT [33]; and Candidate Gene Design, where it generates candidate sequences up to 4 kb in length with transcriptional activity (7/7 candidates) and protein expression (2/7 candidates), while further supporting function‐guided generation of specific gene families such as NLRs. In addition, as a foundation model, we also observed broad improvements of PlantGFM on five downstream regulatory tasks. By unifying gene discovery, design, and regulatory analysis, PlantGFM bridges the gap between computational innovation and biological validation, establishing a scalable framework for plant synthetic biology.

## 2 Results

### 2.1 Architecture and Pretraining of PlantGFM

We here introduce PlantGFM, a decoder‐only sequence‐to‐sequence model specifically tailored for long‐context plant genomic modeling. PlantGFM consists of a token embedding layer, a positional encoding module, 16 Hyena blocks, and an output projection head, totaling approximately 220 million parameters (Methods). One innovative point in designing the architecture is that we replaced the Rotary Position Embedding (RoPE) used in its multi‐head attention module from Meta's LLaMA framework [34] with the more efficient Hyena operator [35]. The Hyena operator has been shown to achieve performance comparable to that of multi‐head attention (MHA) across a range of tasks [35]. Unlike MHA, which explicitly computes attention matrices via scaled dot‐product operations between query and key vectors, the Hyena operator captures both local and global dependencies by leveraging a combination of long‐ and short‐range convolutions along with gating mechanisms. This reduces the time complexity of sequence modeling from O(*n^2^*) to O(*nlogn*) and significantly lowers memory consumption, which enables PlantGFM to model long genomic sequences.

**FIGURE 1:**
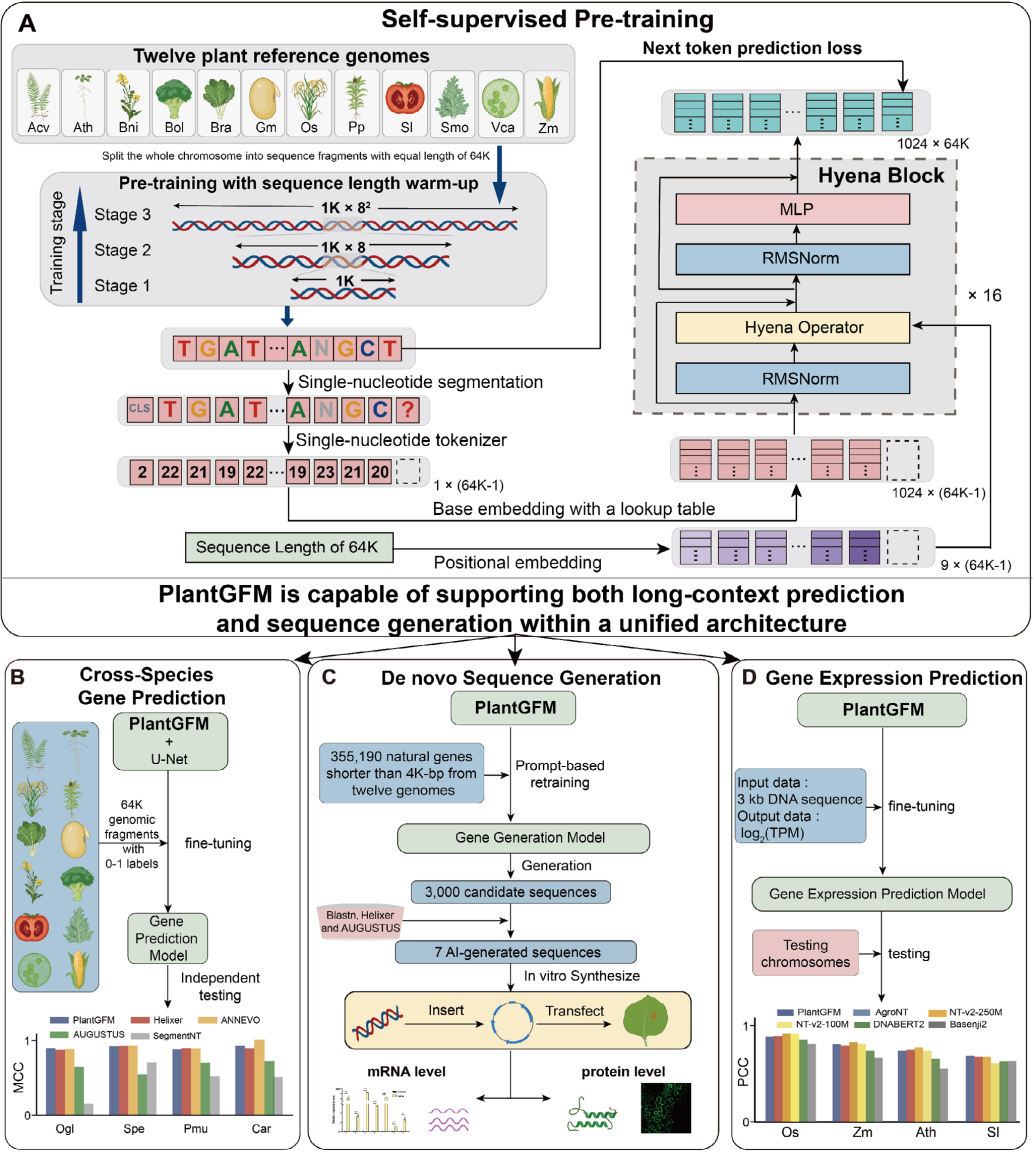
Self‐supervised pre‐training and downstream applications of PlantGFM. (A) Self‐supervised pre‐training. The PlantGFM is trained on reference genomes from 12 plant species, with sequence length warm‐up in three stages (Stage 1, 2, and 3), progressively increasing from 1k to 64k. Each sequence is then tokenized, embedded, and assigned positional encoding, after which it is then passed through 16 Hyena blocks, each containing MLP, RMSNorm, and Hyena operator modules for next‐token prediction. (B) Gene prediction is performed by fine‐tuning the PlantGFM model on 64k genomic fragments with 0–1 labels from ten pre‐trained species. Prediction performance is evaluated using the Matthews correlation coefficient (MCC) on two testing species and four additional lines. (C) Gene generation is performed by re‐training the PlantGFM model on 355,190 plant natural genes shorter than 4 kb with the prompt “gene”. The gene generation model has generated 3,000 candidate gene sequences. These sequences are filtered using Blastn, AUGUSTUS, and Helixer. Seven candidate new genes are selected for synthesis, transfection, and insertion in *N. benthamiana* leaf epidermal cells, with evaluation at the mRNA and protein levels. (D) Gene expression prediction is performed by fine‐tuning the PlantGFM model on some training chromosomes for each species. The input is 3 kb regulatory sequence, and the output is log2(TPM). Testing performance is evaluated using PCC on remaining chromosomes for each species.

Based on this framework, PlantGFM was pre‐trained on the complete genome sequences of 12 model plants (a total of 10.84 billion nucleotides, top of Figure 1A and Table S1) with an unlabeled, self‐supervised and autoregressive manner. To address the challenge of training stability when pre‐training directly on long 64 kb sequences [36], we employed a length warm‐up strategy, which started with short 1 kb fragments in stage 1, gradually increasing to 8 kb in stage 2, and eventually expanded to long 64 kb sequences in stage 3 (Figure 1A, middle). This staged training approach not only effectively mitigated instability during pre‐training but also accelerated convergence (see Methods: 16 epochs in this study). During pre‐training, each sequence fragment was tokenized at the single‐nucleotide level, embedded with positional information (Figure 1A, bottom), and then input into the Hyena blocks. Moreover, we adopted a next‐token prediction objective, enabling PlantGFM to model long‐range dependencies and endowing it with generative capabilities for tasks such as full‐sequence generation and completion.

Benefiting from a state‐of‐the‐art architecture and a robust training strategy, PlantGFM not only delivers improved computational efficiency over traditional Transformer models but also enhances overall performance and training stability. This dual advantage enables scalable modeling of long genomic sequences, supporting both the discovery of known genes (e.g., recovering known natural genes, Figure 1B) and the creation of new knowledge (e.g., *de novo* sequence generation, Figure 1C). This generative capability would be a transformative tool for synthetic biology and genetic engineering in plants. In addition, PlantGFM exhibits high efficiency in traditional regulatory genomics applications, including gene expression prediction (Figure 1D).

### 2.2 Cross‐Species Gene Prediction

**FIGURE 2:**
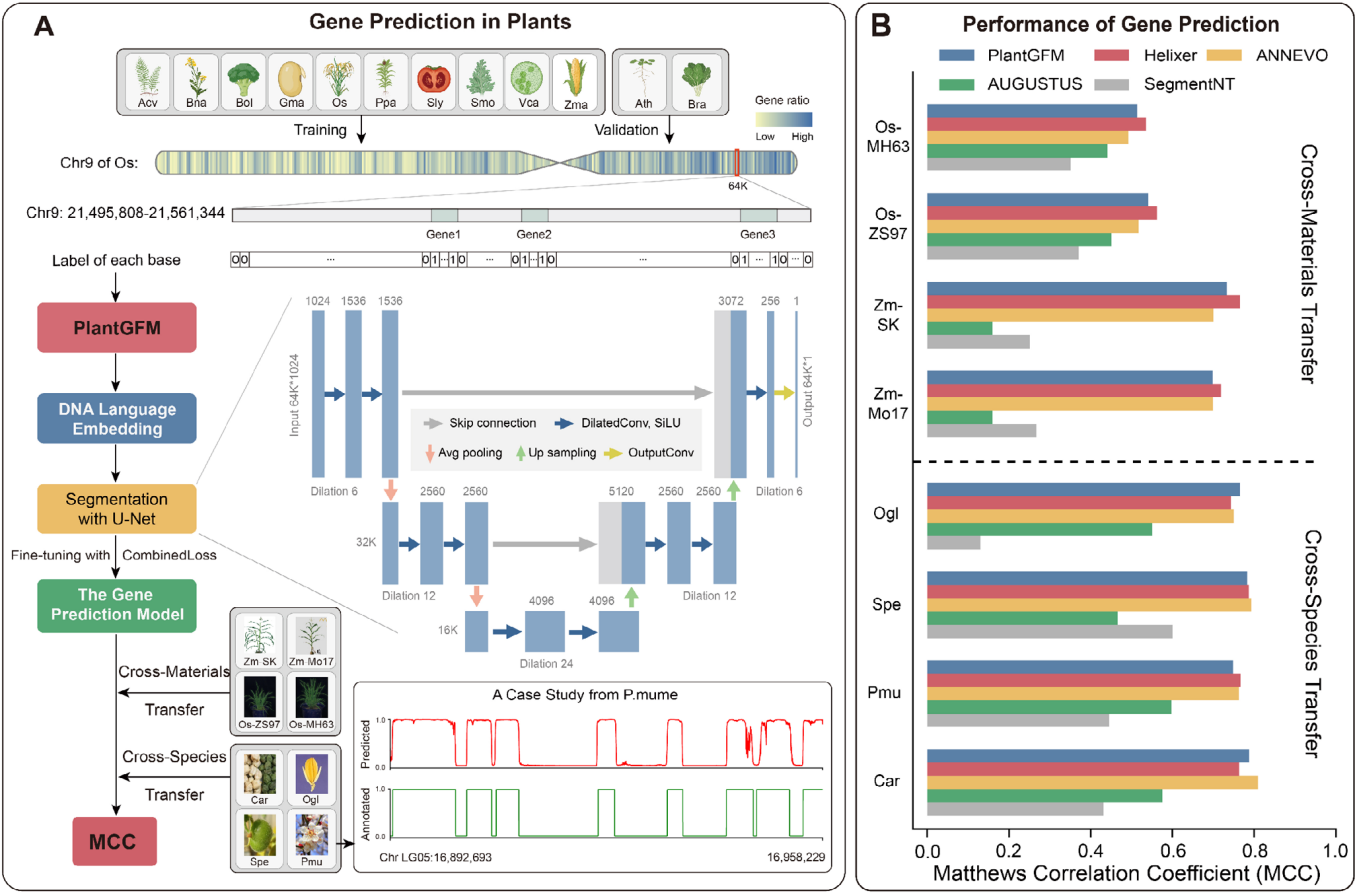
Gene Prediction Using PlantGFM and Comparison with Existing Models. (A) Overview of the gene prediction workflow. Ten model plant species are used to train gene prediction model and two for validation. Chromosome 9 of *Oryza sativa* (Os) is shown as an example, with gene regions labeled as 1 for gene regions and 0 for intergenic regions. The gene prediction model comprises PlantGFM followed by a U‐Net for segmentation. The models were evaluated on 8 plant species or lines divided into two datasets: cross‐materials and cross‐species transfer. At the bottom, an example from Chromosome LG05 of *Prunus mume* (Pmu) is shown, where the predicted gene regions (green) are compared with annotated gene regions (red) based on GFF files. (B) Performance comparison of gene prediction models. The performance of PlantGFM was benchmarked against four existing gene prediction models: Helixer, ANNEVO, AUGUSTUS, and SegmentNT. The results of cross‐materials and cross‐species transfer datasets are presented.

We first explored the ability of PlantGFM, to recover natural genes across multiple plant species, namely evaluating its capability to discover gene regions annotated by the reference genomes at single‐nucleotide resolution. To this end, we selected ten species from the twelve used in pre‐training to construct the fine‐tuning dataset, while the remaining two—*A. thaliana* (Ath), *B. rapa* (Bra)— were designated as a validation set for selecting optimal hyperparameters and checkpoints during fine‐tuning (Figure 2A top).

Using the nineth chromosome of rice as an example of display, we divided it into 351 genomic segments, each of 65,536 bp (64 kb) in length. Then based on the reference genome of IRGSP‐1.0 for *O. sativa ssp*. *japonica* cv. Nipponbare, each nucleotide within these fragments was labeled as follows: nucleotides corresponding to the gene region were labeled as “1”, while all others were labeled as “0” (Figure 2A, top). Building on this setup, we developed a gene prediction model based on PlantGFM, inspired by the design of SegmentNT [33], which incorporates a U‐Net [37] (Figure 2A, middle). In the detailed process of data flow, each genomic fragment first undergoes context‐dependent DNA semantic feature extraction through PlantGFM, forming its high‐dimensional language embeddings. These embeddings are then fed into the U‐Net, which performs nucleotide‐level predictions of “0” or “1” (Figure 2A, middle). Finally, considering the imbalance between gene regions and intergenic regions in plants, we designed a loss function by combining Binary Cross‐Entropy (BCE) [38] and Intersection over Union (IoU) [39].

To evaluate the generalization capability of the gene prediction model, we designed a two‐step independent testing strategy: the first step involved cross‐material (i.e., intra‐species transfer across different cultivars or accessions within the same species) transfer testing using four materials from two species included in the pre‐training corpus—Os‐MH63 (Oryza sativa Minghui 63), Os‐ZS97 (Oryza sativa Zhenshan 97) [40], Zm‐SK (Zea mays Shenkang) [41] and Zm‐Mo17 (Zea mays Mo17) [42]; the second step consisted of cross‐species transfer testing covering four plant species: *O. glaberrima* (Ogl), *S. pennellii* (Spe), *P. mume* (Pmu), and *C. arietinum* (Car). None of the tested materials or species were involved in the model's pre‐training or fine‐tuning processes.

Specifically, for each material or species involved in the testing, we slice its genome into equal‐length 64 kb fragments and then feed them to the gene prediction model. We evaluated the model's performance by calculating the consistency between the prediction and the ground truth annotations from the reference genomes, using Matthews Correlation Coefficient (MCC) as a performance metric (Methods). To provide a more intuitive demonstration of the model's performance, we selected a genomic fragment from chromosome LG05 of *P. mume*, as an example (Figure 2A, bottom). The results showed that the predictions were highly consistent with the ground truth annotations. The model successfully identified most gene regions, with only minor deviations observed at a few boundary areas.

In the cross‐material evaluation, our model achieved MCC values of 0.541 and 0.514 for Os‐ZS97 and Os‐MH63, respectively, while attaining 0.733 for Zm‐SK and 0.698 for Zm‐Mo17 (Figure 2B, top). When compared to existing methods, the model demonstrated consistent improvement over ANNEVO [19], AUGUSTUS, and SegmentNT across all tested materials, though it slightly trailed Helixer. This performance pattern was further corroborated in the cross‐species evaluation. The MCC values achieved by PlantGFM across the four species—*O. glaberrima*, *S. pennellii*, *P. mume*, and *C. arietinum—*were 0.765, 0.783, 0.748, and 0.788, respectively, yielding a mean MCC of 0.771. This performance surpassed that of Helixer (mean MCC: 0.765) and greatly exceeded both AUGUSTUS (0.548) and SegmentNT (0.402), while remaining competitive with ANNEVO (0.779) (Figure 2B, bottom). It implies that PlantGFM generalizes well across divergent species and performs robustly in non‐model plant genomes.

In addition, we also evaluated the performance of PlantGFM in predicting two key genomic elements—exon and CDS, because accurate prediction of these two regions enables complete inference of the entire gene structure. Specifically, using the same training approach as in the gene prediction task, we trained separate models for exon and CDS prediction and tested them on the aforementioned eight species (note that the exon prediction task involved only seven species, as the annotation file for Zm‐SK lacked exon information). As shown in Figure S1A, on the exon prediction task, PlantGFM outperformed ANNEVO, AUGUSTUS, and SegmentNT, while slightly lagging behind Helixer. On the CDS prediction task, PlantGFM surpassed AUGUSTUS, approached the performance of Helixer, but still lagged behind ANNEVO (Figure S1B).

These results above illustrate the balance between specialization and generality achieved by PlantGFM. AUGUSTUS predictions depend heavily on external evidence such as RNA‐seq but fails to match the transferability of end‐to‐end deep learning models when transferred to new materials or species. SegmentNT, while also derived by fine‐tuning a genomic foundation model (NT‐v2‐250 m), was biased toward mammalian gene architectures and thereby limiting its applicability to plant genomes (Figure 2B). For the two accurate tools, Helixer and ANNEVO are vertically specialized expert models that have been carefully optimized for gene prediction within their target domains, and consequently achieve state‐of‐the‐art accuracy in our comparisons (Figure 2B). Nevertheless, PlantGFM consistently ranks second in both cross‐material and cross‐species benchmarks, attaining performance comparable to two expert models while substantially outperforming AUGUSTUS and SegmentNT. In the following sections, we will demonstrate the stronger generality that PlantGFM possesses—but that Helixer and ANNEVO lack—and further showcase its outstanding performance across multiple domains, including de novo sequence design, regulatory element prediction, and several other tasks.

### 2.3 Importance of Long‐Context Input and Single‐Nucleotide‐Resolution on Modeling

To further investigate why PlantGFM achieves performance comparable to current state‐of‐the‐art expert models in the gene prediction task and to determine which components played significant roles, we conducted the following ablation analysis on two factors: long‐context input and single‐nucleotide resolution. We selected four representative plant genomic models for comparison: AgroNT [27], PlantDNAMamba [28], PlantCAD [43], and GPN [18]. The first two support longer context windows (6 kb for AgroNT, 2 kb for PlantDNAMamba) but employ k‐mer tokenizers; the latter two have shorter context windows (both 600 bp) and use single‐nucleotide tokenizers. To ensure a fair comparison and maintain a consistent number of input tokens across all five models during training, we selected a context window of 60 kb — a length evenly divisible by the context window sizes of all four baseline models. Therefore, we first randomly selected 20,000 sequences (each of 64 kb) from the training set of this task, and uniformly trimmed them to a length of 60 kb. Using the same dataset, we performed full‐parameter fine‐tuning on five plant genomic foundation models—AgroNT, PlantDNAMamba, PlantCAD, GPN, and PlantGFM (our model both have these two factors) —and evaluated their performance on the same validation set after fine‐tuning (Table S2).

As shown in Figure S2, PlantGFM achieved the best performance (MCC = 0.764) among the five models, owing to its combined use of a single‐nucleotide tokenizer and 64 kb long‐context modeling. Although PlantCAD and GPN were pre‐trained with shorter context lengths (both 600 bp), they also performed relatively well (MCC = 0.753 and 0.721, respectively) due to their use of single‐nucleotide tokenizers. In contrast, AgroNT and PlantDNAMamba, which were trained with k‐mer tokenizers, exhibited inferior results compared to PlantCAD and GPN—despite having longer pre‐training contexts (AgroNT: 6 kb; PlantDNAMamba: 2 kb). These findings demonstrate that long‐context modeling and single‐nucleotide resolution are both crucial for enhancing performance in the gene prediction task.

### 2.4 De Novo Gene Generation

One of the most striking breakthroughs in AI in recent years is generative AI, which essentially creates new knowledge by fully leveraging its powerful emergent capabilities on top of a deep mastery of existing knowledge [21, 44, 45]. In view of this, our ultimate goal is definitely to investigate whether genome language models can generate novel plant genes with specified functions and enhanced functional performance. Although the latest Evo 1.5 [26] partially achieves this by leveraging the clustering of functional genes in prokaryotic genomes, it remains unknown whether this strategy will be effective for plant genomes, which contain a large number of transposon insertions. Here, our goal was to first test whether completely AI‐generated DNA sequences—without any natural homologs—can be recognized, transcribed, processed, and in some cases translated by plant cellular machinery.

**FIGURE 3:**
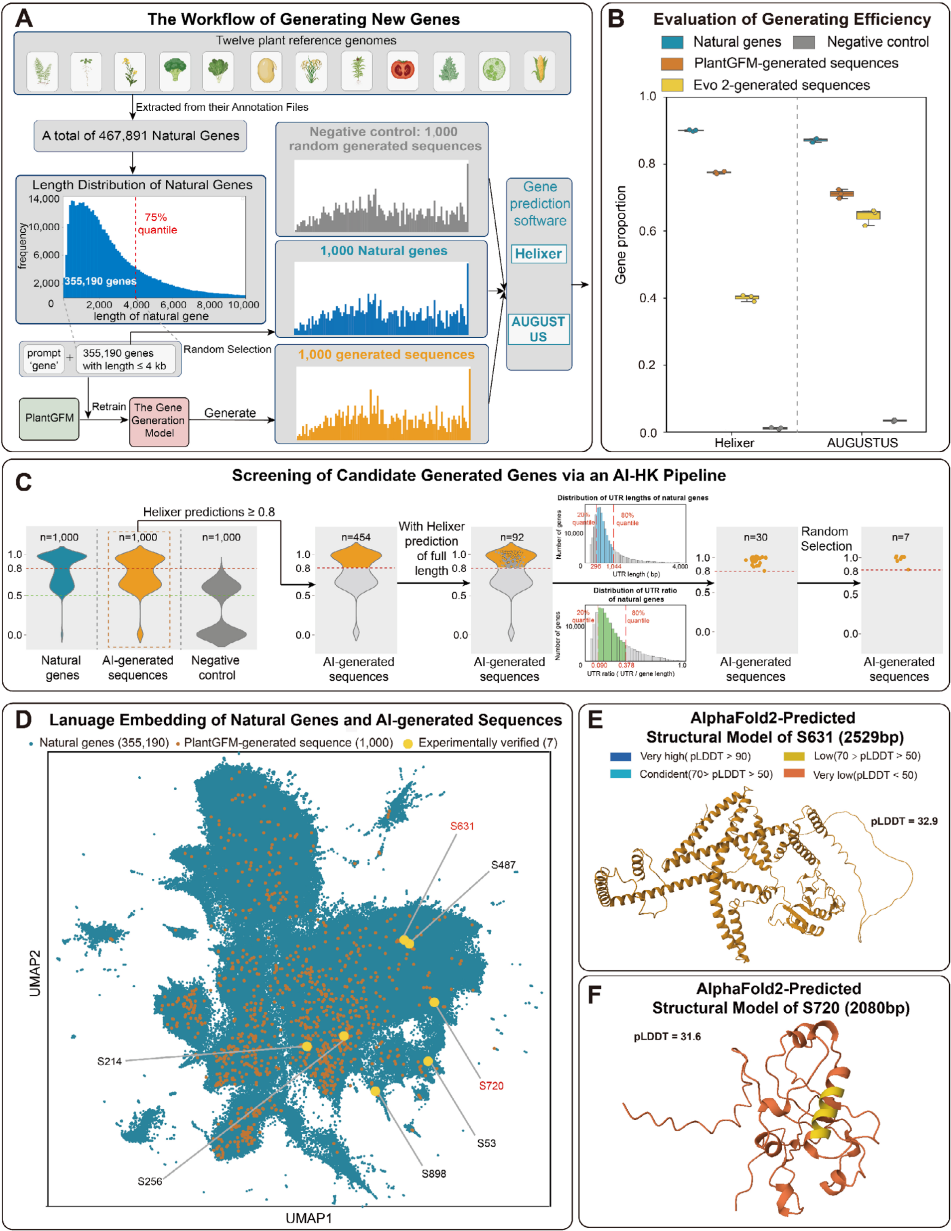
*De novo* Generation and Evaluation of New Genes. (A) The Workflow of Generating New Genes. A total of 467,891 natural genes from 12 plant reference genomes are used. Genes shorter than 4 kb are selected (355,190 genes) and used to retrain the PlantGFM model with the prompt “gene”. The model generates 1,000 new genes, which are compared to two control sets: one consisting of 1,000 randomly generated sequences (negative control) and the other of 1,000 natural genes (positive control). These sequences are annotated using Helixer and AUGUSTUS gene prediction software. (B) Evaluation of Gene Generation Efficiency. The proportion of genes predicted correctly for positive control (natural genes), sequences generated by PlantGFM and Evo 2, and negative control (random sequences), as evaluated by Helixer and AUGUSTUS. (C) Screening of candidate PlantGFM‐generated genes via the AI‐HK pipeline. A multi‐step screening was performed to identify promising PlantGFM‐generated gene candidates. From 1,000 initial sequences, those with Helixer prediction scores ≥ 0.8 were retained (n = 454). Sequences predicted as full‐length genes were further selected (n = 92). Based on the 20th–80th percentile ranges of 5′ UTR length (296–1044 bp) and UTR ratio (0.09–0.38) of natural genes, 30 candidates were chosen, and 7 were randomly selected for experimental validation. (D) Language Embeddings of Natural and Generated Genes. UMAP projection showing the language embeddings of 355,190 natural genes (blue), 1000 PlantGFM‐generated genes(orange), and 7 experimentally verified PlantGFM‐generated genes (light yellow). (E) AlphaFold2‐Predicted Structural Model of S631 (2529 bp). Predicted 3D protein structure for PlantGFM‐generated gene S631, colored by pLDDT confidence scores. (F) AlphaFold2‐Predicted Structural Model of S720 (2080 bp). Predicted 3D protein structure for PlantGFM‐generated gene S720, colored by pLDDT confidence scores.

Our main idea is to re‐train PlantGFM with prompt‐based fine‐tuning using the natural genes from 12 plant species that participated in pre‐training, hoping that the model will learn the patterns and structures of natural genes, and thereby could generate entirely new plant candidate genes (Figure 3A). Specifically, we first extracted the DNA sequences of all 467,891 natural genes from the 12 reference genomes. By observing the length distribution of them, we found that the majority (75%) of them are less than 4 kb in length (Figure 3A, middle left). To ensure that the generated sequences will be more consistent with the lengths of natural genes, we selected those 355,190 natural genes with length less than or equal to 4 kb and added the prompt of “gene” to each. These gene sequences along with their prompts were then fed into PlantGFM and performed two epochs of re‐training, resulting in a Sequence Generation Model (SGM).

Based on SGM, we will begin the work of *de novo* design of new sequences. Specifically, we first used SGM to create a total of 3,000 generated sequences in three batches, with 1,000 sequences per batch, and then apply existing tools [31, 32, 46] to perform screening of the PlantGFM‐generated sequences. To assess the biological plausibility of them, we designed a comparative experiment in which PlantGFM‐generated sequences, Evo 2 ‐generated sequences (another well‐known DNA sequence generation model as an AI positive control), a set of 1,000 natural genes (natural positive control with similar length distribution) (Figure 3A, middle right) and a set of 1,000 purely random sequences (negative control) into two commonly used gene prediction software tools of Helixer [46] and AUGUSTUS [32], and compared their predicted gene proportions. The prediction results showed that the proportion of genes in the PlantGFM‐generated sequences (0.75 using Helixer and 0.70 using AUGUSTUS) was relatively close to that of natural genes (0.9 using Helixer and 0.85 using AUGUSTUS), and substantially higher than that observed for Evo 2‐generated sequences (0.40 by Helixer and 0.65 by AUGUSTUS) as well as the negative control, which yielded near‐zero predictions (Figure 3B). This suggests that the sequences generated by AI have a 75%‐80% probability of aligning with existing tools.

To further validate the novelty of these PlantGFM‐generated sequences, we performed sequence similarity comparisons with BLAST between natural genes from 12 plant species and them. Among the PlantGFM‐generated sequences certified by Helixer (767 in total), only 0.13% (1 new sequence) showed more than 10% nucleotide sequence similarity to existing natural genes, and among those certified by AUGUSTUS (703 in total), no new sequences exceeded 10% similarity. These results strongly suggest that these sequences represent de novo designs rather than recombinations of known genes.

To validate the biological plausibility of PlantGFM‐generated sequences, we analyzed multiple gene‐like properties based on Helixer annotations. Across all metrics, PlantGFM sequences match closer natural genes than Evo 2, including GC content and codon usage, intron counts and total intron lengths (Figure S3), and conserved 5′ donor and 3′ acceptor splice site motifs (Figure S4). This suggests that the generative model not only replicates the base composition characteristics of natural genes but also demonstrates a high degree of plausibility in functionally important coding properties.

### 2.5 Screening of Candidate Generated Genes via an Al‐HK Fusion Pipeline

Following the preliminary evaluation of PlantGFM‐generated sequences, we next integrate generative AI with accumulated biological knowledge for screening pipeline, aiming to provide more reliable candidate sequences for subsequent biological validation. This pipeline was designed as an early attempt to establish a practical workflow that combines computational generation with biological plausibility checks, enabling the discovery of rare but promising candidates for downstream experimentation.

While conducting comprehensive and objective experimental verification on all 1,000 PlantGFM‐generated sequences would be the ideal approach, current DNA synthesis technology presents cost constraints: synthesizing short DNA fragments under 300 bp is economically feasible, whereas producing sequences longer than 1 kb remains prohibitively expensive [8, 47]. Therefore, it is necessary to prioritize the most promising plant gene candidates from the initial 1,000 sequences—reducing the pool to a few dozen—before proceeding with experimental validation.

We developed an AI‐HK (Human Knowledge) fusion pipeline that filters sequences based on two criteria derived from Helixer software: the predicted probability score of each base falling within a gene region, and the reasonableness of predicted UTR length (Figure 3C). First, for each of the 1,000 PlantGFM‐generated sequences, we used Helixer to output a probability score for every base position indicating its likelihood of being part of a gene. We then calculated the mean probability score across all bases as the overall gene probability score for that sequence. Since most natural genes exhibited probability scores exceeding 0.8, we applied this threshold for initial filtering, reducing the pool from 1,000 to 454 sequences. Next, because PlantGFM was fine‐tuned exclusively on complete gene sequences (excluding intergenic regions), we reasoned that the PlantGFM‐generated sequences to similarly represent full‐length genes. Applying this additional criterion further refined our selection from 454 to 92 candidate sequences.

After ensuring the PlantGFM‐generated sequences conformed to human‐curated gene structure conventions, we performed a final screening based on their potential for correct protein translation. Extensive literature indicates that appropriate UTR length is critical for translation efficiency, and mutations in UTR regions can significantly impact ribosomal translation [48]. We therefore analyzed UTR length distributions across all natural genes (Figure 3C) and defined the 20th to 80th percentile range as constituting reasonable UTR length. Applying this criterion reduced our candidate pool to 30 sequences. From these final candidates, we randomly selected 7 sequences for subsequent biological validation of proper transcription and translation (Figure S5 and Table S3).

To further assess the relationship between these candidate sequences and natural genes, we extracted the language embeddings of both the natural 355,190 gene sequences and the 1,000 PlantGFM‐generated sequences using the un‐tuned PlantGFM language model, and performed UMAP dimensionality reduction analysis (Figure 3D) [49]. The results show that the generated genes and the natural genes exhibit similar language grammar, and the 7 experimentally validated AI‑generated sequences are broadly distributed. Consistent with this observation, a quantitative analysis of Maximum Mean Discrepancy (MMD, Methods) [50] in the same embedding space revealed that the distribution of PlantGFM‐generated sequences is closer to that of natural genes than to random sequences (Figure S6).

### 2.6 Experimental Validation of PlantGFM‐Generated Genes

**FIGURE 4:**
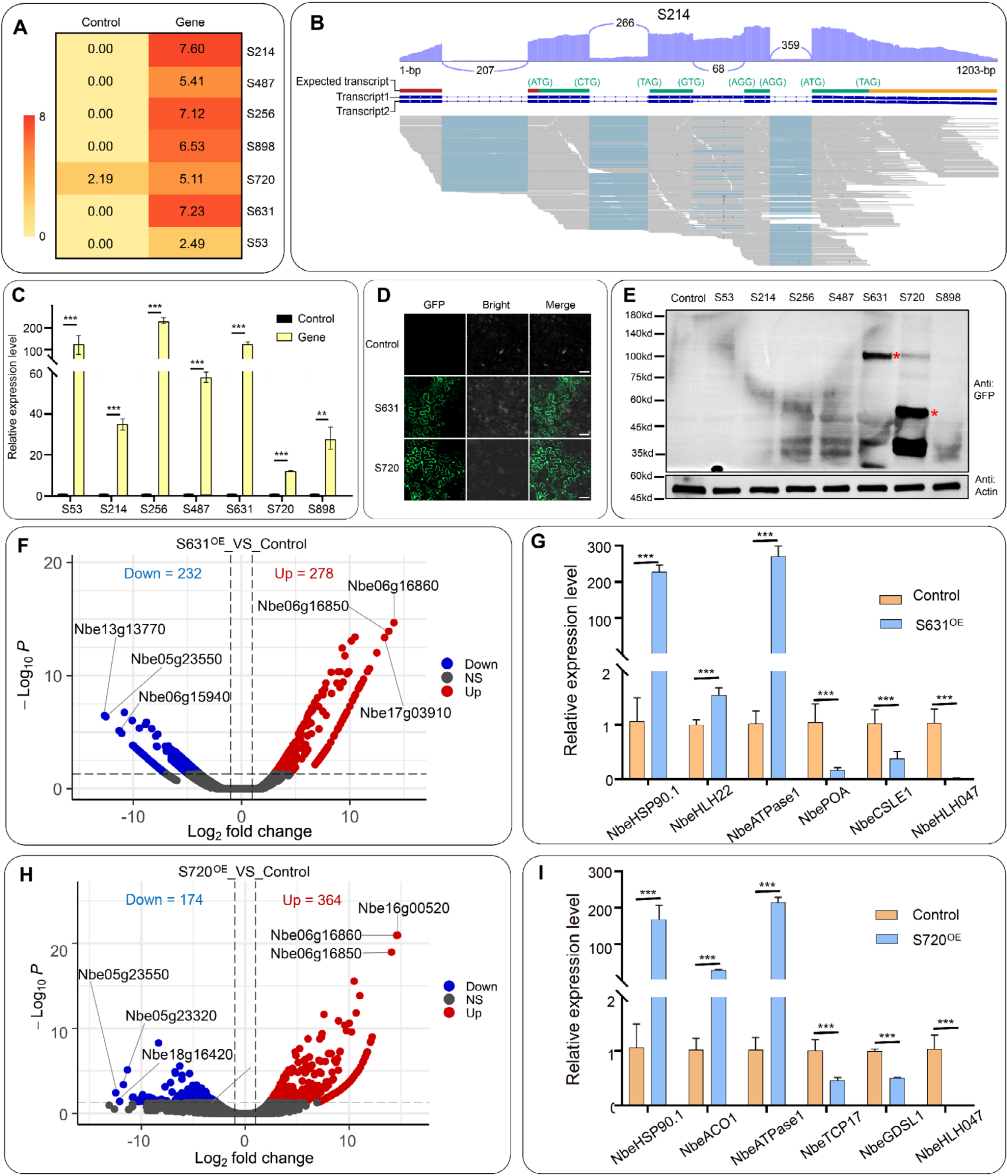
Utilizing the tobacco transient expression system to evaluate the expression and regulatory effects of novel genes. (A) Heatmap of genes expression fold changes analyzed by RNA sequencing. (B) The IGV map of RNA‐Sequencing data of *S214*. The numbers 207, 266, 68, and 359 represent the count of reads undergoing intron splicing and the original sequence and the transcripts have been indicated. The red box represents the 5' UTR, the green box represents the exon, and the yellow box represents the 3' UTR. (C) RT‐qPCR Analysis. *NbEF1α* as the reference gene. Data are presented as means ± SD, and statistical significance was determined using a two‐tailed Student's t‐test. ns, no significance; *p < 0.05; **p < 0.01; ***p < 0.001. (D), (E) Transient‐expression assay. GFP‐tagged novel genes were agro‐infiltrated into Nicotiana benthamiana leaves; After 48 h, GFP fluorescence was imaged with a super‐resolution confocal microscope (D; bar = 50 µm) and total proteins were analysed by western blot using anti‐GFP antibody (E). Anti‐Actin signals verified equal loading. Empty‐vector controls are shown, and the target band is indicated by a red asterisk. (F) Volcano plot showing differential gene expression between the S631 overexpression group and the control group. The x‐axis indicates log_2_ fold change, and the y‐axis indicates –log_10_(p‐value). Red dots represent significantly upregulated genes (p < 0.05, log_2_FC > 1), blue dots indicate significantly downregulated genes (p < 0.05, log_2_FC < –1), and gray dots indicate non‐significant changes. G, I) RT‐qPCR Analysis. *NbEF1α* as the reference gene. Data are presented as means ± SD, and statistical significance was determined using a two‐tailed Student's t‐test. *** p < 0.001. H) Volcano plot showing differential gene expression between the S720 overexpression group and the control group. Significance thresholds and color coding are as described in F.

To determine whether the seven AI‐designed sequences can be recognized, transcribed, processed, and in some cases translated by plant cellular machinery rather than computational artefacts, we subjected them to experimental validation. All full‐length PlantGFM‐generated sequences (Table S4), synthesized *de novo*, were individually cloned into the pFGC5941 expression vector and transiently expressed into *N. benthamiana* leaf epidermal cells, with the empty vector served as the negative control (hereafter referred to as ‘Control’). Transcriptional competence was rigorously quantified by strand‐specific RNA‐seq and independently validated by RT‐qPCR. As the results, all AI‐designed sequences exhibited robust expression, with mean normalized read counts 2‐ to 7‐fold higher than control (Figure 4A). Notably, the S214 locus produced multiple discrete transcript isoforms (Figure 4B), indicative of accurate splice‐site recognition and processing. RT‐qPCR corroborated these findings, revealing 11‐ to 220‐fold increases in steady‐state mRNA levels relative to controls (Figure 4C). Collectively, the data demonstrate that plant cells faithfully transcribe, splice and accumulate mRNAs from PlantGFM‐generated templates, providing empirical evidence that these sequences are not computational artefacts. That alternative splicing of S214 provides indirect but meaningful evidence that the model has learned internal gene structure beyond simple binary distinctions.

To further verify that transcripts from PlantGFM‐generated sequences can be accurately translated, we fused GFP‐Tag to their C‐termini and performed transient expression assays in N. benthamiana. Figure 4D shows that, relative to the control, S631 and S720 produced intense GFP fluorescence, indicating robust accumulation of the corresponding fusion proteins. Immunoblot analysis of total protein extracts detected specific bands at the predicted molecular weights exclusively in S631 and S720, with no signals observed for the remaining sequences, confirming that these two constructs are correctly and stably translated (Figure 4E). Results were reproducible across three independent biological replicates, with the other two shown in Figure S7.

Next, we assessed the transcriptomic impact of S631 or S720 overexpression in tobacco. Volcano plots revealed 278 up‐ and 232 down‐regulated genes in S631^OE^, and 364 up‐ and 174 down‐regulated genes in S720^OE^, relative to empty‐vector controls (Figure 4F,H; Tables S4 and S5). RT‐qPCR confirmed the RNA‐seq change for selected genes (Figure 4G,I). Remarkably, 162 DEGs (Figure S8 and Tables S5 and S6) were concordantly regulated in both lines, including up‐regulation of the heat shock protein NbeHSP90.1 and down‐regulation of the transcription factor NbeHLH047—an overlap consistent with the adjacent UMAP embedding of S631 and S720 (Figure 3D) and indicative of partially shared downstream networks.

In conclusion, we demonstrated that not all arbitrary sequences can be transcribed, correctly spliced, or folded into stable polypeptides and a subset of PlantGFM‐generated sequences was stably translated into detectable proteins, with additional structural support from AlphaFold2 predictions (Figure 3E,F), suggesting a degree of biological plausibility.

### 2.7 Function‐Guided Generation of NLR Genes

**FIGURE 5:**
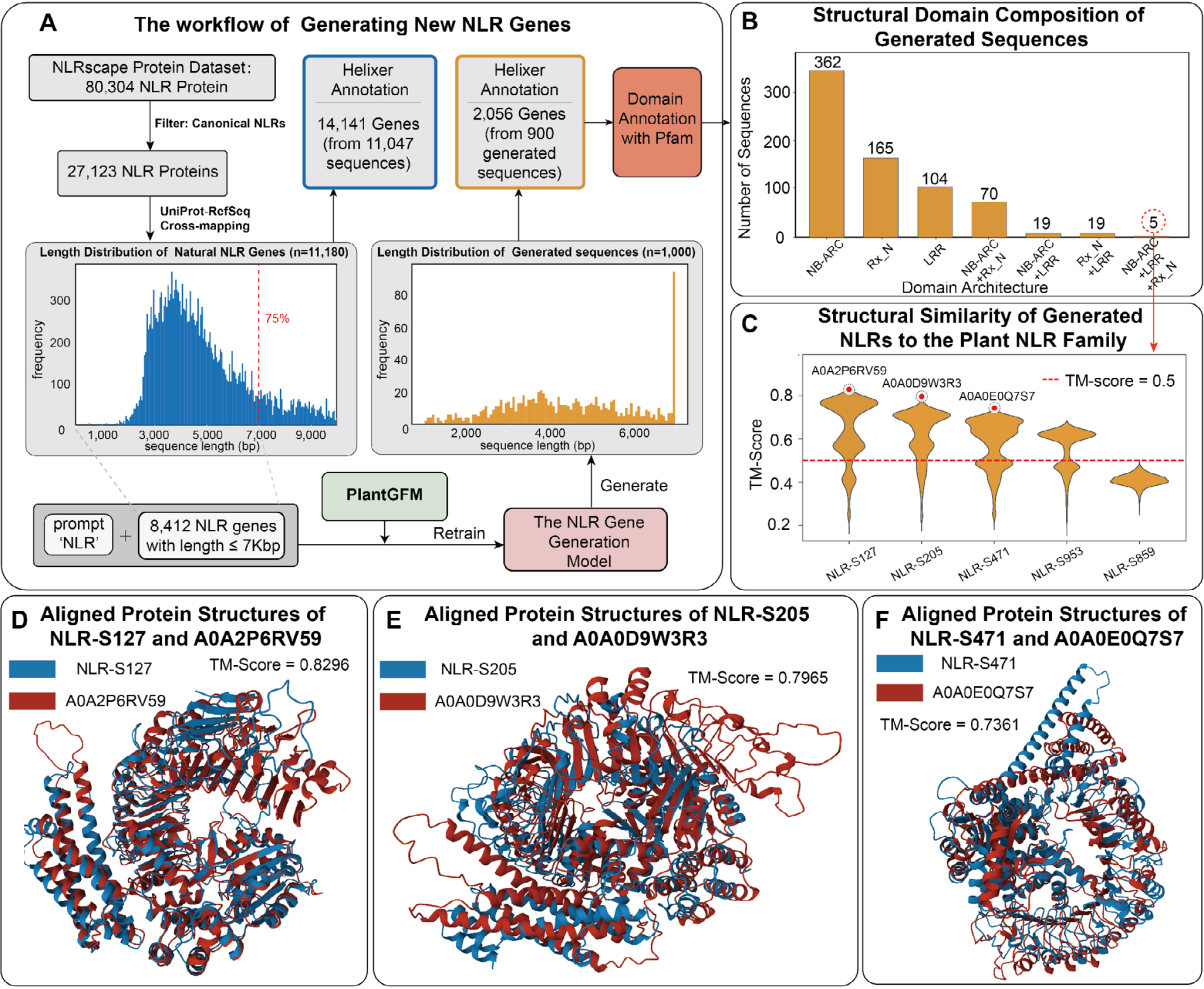
Function‐guided generation and structural characterization of plant NLR genes using PlantGFM. (A) Workflow of NLR gene generation. Canonical NLR proteins were selected from the NLRscape dataset (n = 27,123) and mapped to UniProt–RefSeq to retrieve corresponding genomic sequences (n = 11,184). Genes shorter than 7 kb (n = 8,412) were used to retrain PlantGFM with the prompt “NLR,” yielding the NLR Gene Generation Model. A total of 1,000 sequences were generated, annotated with Helixer, and analyzed for domain composition using Pfam. (B) Structural domain composition of PlantGFM‐generated NLR sequences. The number of sequences containing NB‐ARC, Rx_N, and LRR domains is shown. Five sequences exhibited a complete canonical NLR architecture containing all three hallmark domains. (C) Structural similarity of generated NLR candidates to the plant NLR family. TM‐scores of the five canonical candidates compared to the reference set of 27,123 natural NLR proteins are shown as violin plots, with the commonly accepted TM‐score threshold of 0.5 indicated by the dashed line. (D–F) Aligned protein structures of top generated NLR candidates with their best‐matched natural templates. Superimpositions of NLR‐S127 with A0A2P6RV59 (D, TM‐score = 0.8296), NLR‐S205 with A0A0D9W3R3 (E, TM‐score = 0.7965), and NLR‐S471 with A0A0E0Q7S7 (F, TM‐score = 0.7361) are shown, illustrating highly similar overall folds between generated sequences (blue) and natural templates (red).

Building on this proof of biological plausibility, we next evaluated PlantGFM's emergent ability to generate specific biological architectures through function‐guided gene generation. To this end, we focused on the Nucleotide‐binding Leucine‐rich Repeat (NLR) gene family as a representative case. A dataset of 80,303 plant NLR proteins was initially retrieved from NLRscape, an atlas of plant immune receptors curated via profile Hidden Markov Models [51]. To ensure that PlantGFM could capture the full sequence grammar of NLRs, only canonical NLRs (*n* = 27,123) containing complete coiled‐coil (CC), nucleotide‐binding (NB‐ARC), and leucine‐rich repeat (LRR) domains were retained. These protein sequences were mapped to the UniProt–RefSeq database [52, 53] to retrieve their corresponding genomic DNA sequences, yielding a total of 11,184 NLR genes. Analysis of gene length distribution showed that approximately 75% of natural NLR genes were shorter than 7 kb. Based on this, we selected 8,412 NLR genes shorter than 7 kb, together with the prompt “NLR,” to retrain PlantGFM, ultimately yielding the NLR Gene Generation Model (NLR‐GGM, Figure 5A).

Using NLR‐GGM, we generated 1,000 sequences and then annotated them with Helixer. As a result, 900 sequences were annotated as to contain predicted genes, totaling 2,056 genes. The predicted coding sequences were translated into protein sequences and subsequently analyzed for domain composition using the Pfam database [54]. As shown in Figure 5B, PlantGFM‐generated NLR candidates were partly annotated to have multiple immune‐related domains, including NB‐ARC (n = 362), Rx_N (n = 165; N‐terminal coiled‐coil–like domain found in certain NLR proteins), and LRR (n = 104), consistent with the canonical domain composition of NLR proteins. This demonstrates that NLR‐GGM has captured the sequence semantics and domain grammar of the key NLR domains.

Notably, five candidate sequences exhibited a complete canonical NLR architecture, containing all three hallmark domains: Rx_N, NB‐ARC, and LRR, and were selected for the following analysis. We next evaluated the sequence and structural properties of the five canonical NLR candidates. On one hand, BLASTN comparisons against natural NLR genes (11,184 natural NLR genes) revealed that none of the candidates shared more than 10% nucleotide sequence identity, indicating substantial sequence novelty. On the other hand, 3D structure predictions using AlphaFold2, coupled with comparisons against a reference set of 27,123 natural canonical NLR proteins, showed that four out of the five candidates exhibited similar overall folds, with TM‐scores above the commonly accepted threshold of 0.5 (Figure 5C). Structural alignment of the top three candidates (NLR‐S127, NLR‐S 205, and NLR‐S471) with their best‐matched natural structural templates revealed highly similar overall folds (Figure 5D–F).

Overall, we demonstrate PlantGFM's emergent ability to generate highly novel NLR gene candidates in plants while preserving canonical domain architectures and biologically plausible 3D folds. By capturing specific sequence grammar and structural constraints, it is expected to become a powerful tool for function‐guided design of plant genes.

### 2.8 Regulatory Genomics Applications of PlantGFM

Beyond gene prediction and generation, we finally assessed PlantGFM's performance on five core regulatory genomics tasks relevant to transcriptional regulation: gene expression prediction, chromatin accessibility (open chromatin regions, OCRs) prediction, TFBSs prediction, CREs strength prediction, and a zero‐shot task of Variant Effect Prediction (VEP).

**FIGURE 6:**
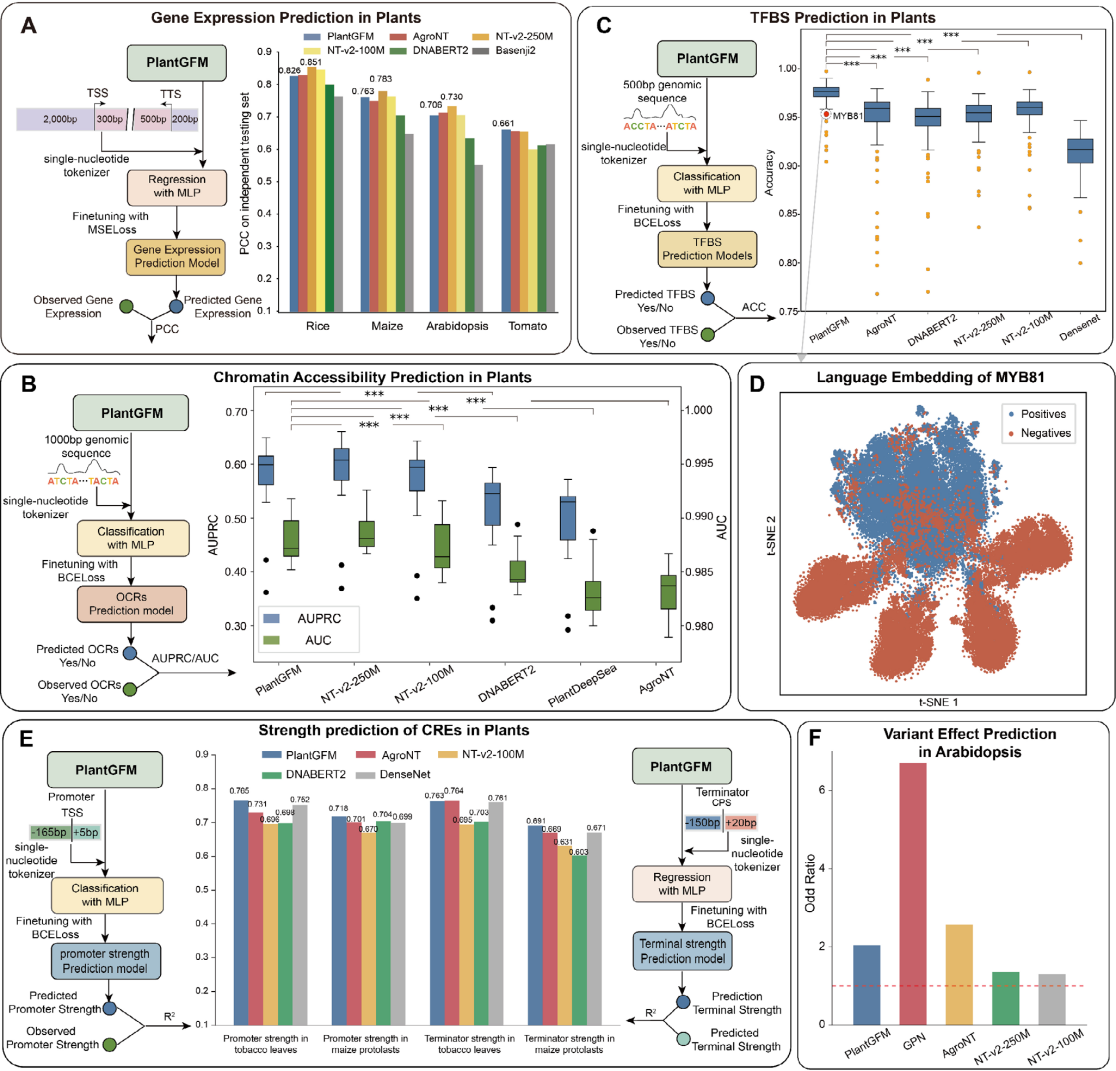
Multi‐task Applications of PlantGFM in Plant Regulatory Genomics. (A) Gene Expression Prediction. PlantGFM was fine‐tuned to predict expression levels using TSS‐to‐TTS sequences. Following single‐nucleotide tokenization and MLP‐based regression, the model was optimized via MSELoss. Performance (PCC) is compared across species against state‐of‐the‐art gLMs, including AgroNT and Basenji2. (B) Chromatin Accessibility Prediction. Evaluation of PlantGFM and other gLMs (e.g., PlantDeepSEA) in predicting accessibility within 1 kb genomic regions, measured by AUPRC and AUC. The box plots illustrate the distribution of AUPRC and AUC values across 19 independent maize tissue datasets, where each data point represents a specific tissue. (C) TFBS Prediction. Comparison of PlantGFM, TSPTFBS2.0, and baseline gLMs for transcription factor binding site (TFBS) classification using 500 bp sequences across *Z. mays*, *O. sativa*, and *A. thaliana*. The box plots represent the performance distribution across 104 distinct TFBS datasets (including 96 maize, 7 rice, and 1 Arabidopsis TFs), with each point indicating an individual TF dataset. (D) Language Embedding of MYB81. t‐SNE visualization showing the separation of positive and negative TFBS samples in the embedding space. (E) Strength Prediction of CREs in Plants. Fine‐tuning of PlantGFM for predicting promoter and terminator functional strengths using MLP‐regression. Performance is evaluated by R^2^ in tobacco and maize systems against four representative models. (F) Variant Effect Prediction in Arabidopsis. Assessment of PlantGFM's capability in predicting the impact of genetic variants, with performance quantified by Odd Ratio. Statistical significance between PlantGFM and baseline models was evaluated using a two‐sided Wilcoxon signed‐rank test (*p < 0.05; **p < 0.01; ***p < 0.001).

For gene expression, since the main function of regulatory sequences is to determine the abundance of gene expression, we employed an existing dataset composed of 3 kb‐long sequences to train and evaluate the model (Table S7) [55]. We fine‐tuned PlantGFM with a regression head consisting of a multilayer perceptron (MLP), optimized using mean squared error (MSE) loss. Model performance was evaluated using the Pearson correlation coefficient (PCC) between observed and predicted expression levels (Figure 6A, left). As a product of the LLM era, PlantGFM shows clear advantages over Basenji2, a typical model from deep learning‐era (Figure 6A). In a comparative analysis of other genomic language models—including AgroNT, NT‐v2 [18], and DNABERT2 [17]—NT‐v2 exhibits the strongest overall performance, leading in predictions for rice, maize, and Arabidopsis. PlantGFM achieves optimal results in tomato and remains highly competitive in the other three species, with only marginal differences from NT‐v2 (Figure 6A). Specifically, PlantGFM attained PCC values of 0.826 in rice, 0.763 in maize, 0.706 in Arabidopsis, and 0.661 in tomato, yielding a mean PCC of 0.739. This represents a performance superior to AgroNT (0.738) and NT‐v2‐100 m (0.728), and greatly exceeds DNABERT2 (0.686) and Basenji2 (0.651), though it remains slightly below NT‐v2‐250 m (0.754) that is the world's leading model for prediction tasks at present.

For chromatin accessibility prediction, we fine‐tuned our model on 19 ATAC‐seq datasets from diverse maize tissues [56] (Supplementary Materials). Each genomic region was encoded as a 1,000 bp DNA sequence and used to train a binary classifier composed of an MLP, optimized with binary cross‐entropy loss (BCELoss), to predict whether a region is open or closed (Figure 6B, left). Model performance was evaluated using the area under the receiver operating characteristic curve (AUC) and area under the precision‐recall curve (AUPRC) across tissues (Figure 6B, right). Similarly, when compared to the previously mentioned baselines and the CNN‐based prediction model PlantDeepSEA, PlantGFM achieved mean AUROC and AUPRC values of 0.988 and 0.574, respectively, across 19 tissues. This performance significantly surpassed all baseline models (Figure 6B) and was comparable to NT‐v2‐250 m (mean AUROC: 0.989, AUPRC: 0.585), demonstrating its enhanced generalization capability across tissue‐specific accessibility patterns.

For TFBSs prediction, we selected 104 TFBSs datasets from a total of 389 plant TFBSs, where the prediction accuracy was not perfect (test set ACC < 0.95), based on the results of TSPTFBS2.0 [57] (Supplementary Materials). These included 96 maize TFs, 7 rice TFs, and 1 Arabidopsis TF. As shown in Figure 6C, PlantGFM achieved a mean ACC of 0.973, surpassing TSPTFBS2.0 (mean ACC: 0.914) by a significant margin of 6 percentage points. When compared to other baselines such as NT‐v2‐250 m (mean ACC: 0.955) and AgroNT (mean ACC: 0.944), PlantGFM maintained a clear competitive advantage. To better understand why the model show stronger discriminative capability, we performed t‐SNE projection [58] on the language embeddings of MYB81 (using all samples from both training and test sets). As shown in Figure 6D, the embeddings of positive and negative samples were clearly clustered into distinct groups. This demonstrates that through pre‐training, PlantGFM has effectively captured sequence‐level functional syntax, enabling it to distinguish sequences with different regulatory roles.

In CREs strength prediction task, we utilized plant core promoters datasets from Jores et al. [59] and plant terminators datasets from Gorjifard et al. [60], both assessed via STARR‐seq assays (Table S8). It was illustrated that for promoters measured in two transcription systems, PlantGFM outperformed leading models including NT‐v2‐100 m, DNABERT2, and DenseNet [61], and for terminators measured in two transcription systems, it also outperformed Gorjifard et al.’s DenseNet (Figure 6E). When compared to AgroNT, PlantGFM outperforms in three out of four datasets on plant core promoters and terminators, with a slight lag in one dataset (Figure 6E).

In the task of VEP, we evaluated 2.6 million SNPs from chromosome 1 of *A. thaliana*, sourced from the 1001 Genomes Project [62]. For each single nucleotide variant (SNV), we calculated the difference in log‐likelihood (or log‐pseudolikelihood) between the pre‐ and post‐variant sequences as a score of its deleteriousness. To assess the ability of the models to identify functional variants, we followed the evaluation metric used by GPN [18] and computed the odds ratio of rare variants (allele count AC = 1) versus common variants (allele frequency AF ≥ 5%) in the tail of the deleteriousness score distribution. We selected four representative genomic models for comparison: PlantGFM, AgroNT, NT‐v2‐250 m, NT‐v2‐100 m, and GPN (note: DNABERT2 was excluded as it relies on BPE tokenization and is not applicable to this task). For the results, PlantGFM demonstrates a certain capability in identifying deleterious variants, with an enrichment level comparable to AgroNT and higher than that of NT‐v2‐250 m and NT‐v2‐100 m, yet still lower than that of GPN (Figure 6F), a renowned expert model in this field, which was specifically optimized and trained for this task. This result indicates that, on the VEP task, the capability of genomic language models currently still lags behind that of specialized expert models in this field.

Overall, PlantGFM performs comparably to or exceeds existing methods depending on the evaluation setting across various plant regulatory genomics tasks. Although NT‐v2‐250 m slightly outperforms PlantGFM in gene expression prediction and chromatin accessibility classification, this difference is largely attributable to the scale and diversity of the pretraining data: NT‐v2‐250 m was trained on 850 representative species spanning both prokaryotes and eukaryotes, whereas PlantGFM is based on only 12 reference plant genomes. Nevertheless, PlantGFM surpasses all baseline models in TFBS prediction, highlighting its exceptional ability to capture fine‐grained regulatory grammar at single‐nucleotide resolution. More importantly, compared with BERT‐style encoder models such as NT‐v2 and AgroNT, PlantGFM's greater advantage lies in its native support for de novo sequence generation.

## 3 Discussion

PlantGFM, as the first application of the Hyena operator within a plant‐oriented genomic foundation model, achieves long‐context modeling up to 64 kb with single‐nucleotide resolution. PlantGFM is capable of supporting long‐context prediction and sequence generation within a unified architecture. It can be fine‐tuned for various plant genome prediction tasks and has achieved performance comparable to or exceeds existing methods, eliminating the need for different deep learning architectures and training on specific genome sequences for each task.

A major contribution of this work is the introduction of the Hyena architecture into plant genomics. This allows PlantGFM to act as a chassis model, significantly simplifying the model selection and design process while maintaining high performance. Notably, PlantGFM is hardware‐friendly and can be fine‐tuned on most consumer GPUs (e.g., Nvidia RTX 3090). The key innovation lies in its ability to model genomic sequences with long‐context, enabling AI‐based generation of relatively long DNA sequences (up to 4 kb). We provided the first demonstration of DNA–RNA–protein expression of LLM‐generated genomic sequences in a plant system, proving their feasibility in terms of recognition, transcription, and translation. Although the specific biological functions are yet to be determined, these advances open a new pathway for the *de novo* design of novel genes in plants.

However, a key limitation of this study is that the current data do not allow us to assign specific biological functions to the generated sequences. The observed gene expression changes following overexpression—particularly those involving heat‐shock genes—likely reflect a generalized stress response rather than functional similarity. Any mechanistic or functional inference would require dedicated biochemical and genetic characterization. Additionally, only two out of seven tested sequences accumulated detectable protein, underscoring that achieving robust biological functionality remains a long‐term challenge.

## 4 Conclusion

In summary, PlantGFM represents a significant step forward in plant genomics by integrating the Hyena architecture to support long‐context prediction and sequence generation. While current limitations exist regarding specific functional assignment, the model provides a powerful and accessible tool for the research community.

Future improvements will focus on three key areas: (1) Function‐Guided fine‐tuning: Building on our demonstration of function‐guided NLR gene generation, future work will focus on experimental validations on whether the NLR genes we generated are functional. (2) High‐throughput validation: We aim to develop new plant chassis cell models and high‐throughput screening frameworks to validate thousands of generated sequences efficiently. (3) Scaling and Optimization: We will continue to optimize the model by incorporating more plant species for pretraining, increasing context recognition length, and utilizing Mixture of Experts (MoE) [21] to expand network capacity without significantly increasing computational costs. These efforts aim to evolve PlantGFM into a highly generalizable, broad‐perspective, and lightweight model for plant science.

## 5 Methods

### 5.1 PlantGFM Architecture

PlantGFM is a decoder‐only sequence‐to‐sequence model. In designing its architecture, we followed the Hyena layers architecture from Evo [25], replacing the multi‐head attention module (equipped with Rotary Positional Embeddings, RoPE) in the LLaMA model framework with the more efficient Hyena operator, while retaining the original positions of RMSNorm and the Swish Gated Linear Units (SwiGLU). In terms of specific configurations, PlantGFM consists of 16 Hyena blocks, with an embedding size of 1024, a GLU intermediate size of 3072, and an epsilon value of 1e‐5 for RMSNorm. The total number of model parameters is 220 million.

### 5.2 Hyena Operator

Hyena, introduced by Poli et al., is an operator designed to replace the multi‐head self‐attention (MHA) mechanism in Transformer. It achieves comparable performance to MHA with a computational complexity of $O(L\mathrm{lo}g_{2}L)$ by leveraging short convolutions, implicitly parameterized long convolutions, and gating mechanisms. At the same time, it significantly enhances computational efficiency compared to MHA. Suppose the input is $u\in R^{L\times D}$, where *L* is the sequence length and *D* is the embedding dimension. In Hyena Operator, the model first passes through a linear layer along with a depth‐wise short convolution layer (with *h_s_* as the short convolution kernel) to obtain *x*_1_,*x*_2_, and *v*:

(1) $$z=\mathrm{DWConv}1d\left(h_{s},\;uW\right),\;z\in R^{L\times 3D},\;W\in R^{D\times 3D}$$

(2) $$x_{1},x_{2},v=\mathrm{Split}\left(z\right),\;x_{1},x_{2},v\in R^{L\times D}$$

Next, gating (where * represents element‐wise multiplication) and a long convolution based on the Fast Fourier Transform (FFT) are applied to produce the output *y*:

(3) $$y=x_{2}\;*\mathrm{FFTConv}\left(h_{l},\;x_{1}*v\right),\;y\in R^{L\times D}$$

The convolution kernel for the long convolution *h_l_* is implicitly learned by a small feedforward network (FFN) called “Hyena Filters” which takes the time index as input and optionally incorporates positional encoding:

(4) $$h_{l}=\mathrm{HyenaFilters}\left(\mathrm{PosEncoding}\left(L\right)\right)$$

According to Poli et al.’s research, when the sequence length is 8 k, the Hyena Operator is twice as fast as the highly optimized MHA mechanism, and at a sequence length of 64 k, it is 100 times faster.

### 5.3 Data Preparation and Pre‐Processing

We selected 12 representative plant species, covering both monocotyledonous and dicotyledonous plants, including model species (such as *Arabidopsis thaliana* and rice), major crops (such as soybean and maize), and plants with distinct evolutionary positions (such as *Adiantum capillus‐veneris*). These species differ substantially in terms of genome size, chromosomal architecture, gene density, and ploidy level. Based on this diversity, we expect the model to learn a more diverse “syntax” of plant genomes, thereby significantly enhancing its generalization ability. The genomic data was segmented into fixed‐length sequences of 65,536 bp. During the segmentation process, we introduced a random overlap of 60 to 100 bp between adjacent sequences and filtered out those with an “N” content exceeding 5%, resulting in the construction of a high‐quality training dataset. After data preprocessing, the total number of tokens in the pretraining dataset reached 10.84 billion, which was further divided into training and validation sets based on chromosomes.

### 5.4 Tokenization

For our tokenization strategy, we adopted the single‐nucleotide tokenization method. Our base vocabulary includes the nucleotides “A”, “T”, “C”, “G”, “N”, as well as special tokens such as “<CLS>”, “<SEP>”, and “<PAD>”. Additionally, we reserved 12 tokens, named “<RESERVED1>” to “<RESERVED12>”, for future generative task prompt construction. As a result, the total vocabulary size is 24 (Table S9).

### 5.5 Pre‐Training Strategy

The pre‐training strategy and hyperparameter configuration were both adapted from HyenaDNA [63]. The pre‐training process was divided into three stages, with each subsequent stage initialized using the pre‐trained weights from the previous one. Specifically, in the first stage, we performed pre‐training on short sequences of 1,024 bp, which were extracted from the middle of the full‐length 65,536 bp sequences, until the perplexity on the validation set no longer decreased. The model weights obtained from the first stage were then used to initialize the second stage, which continued training on sequences of 8,192 bp, also extracted from the full‐length sequences. Finally, the third stage completed pre‐training using the full‐length 65,536 bp sequences. The learning rate was set to 6e‐4 for all three stages, with a total of 30,000 training steps per stage. During the first 1,000 steps, the learning rate was linearly increased from 0 to 6e‐4, followed by cosine decay to 0 over the remaining steps. The optimizer used in all stages was AdamW. For the first stage, the beta values were set to *β_1_* = 0.9 and *β_2_* = 0.99, while for the second and third stages, *β_1_* and *β_2_* were set to 0.9 and 0.95, respectively. Additional pre‐training details are provided in Table S10.

### 5.6 Fine‐Tuning Setup

All fine‐tuning data was processed into single‐nucleotide sequences and then fed into the model for fine‐tuning. For gene prediction, we integrated a U‐Net network with two down‐sampling and two up‐sampling layers as the segmentation head at the end of the model. U‐Net is a neural network architecture designed for image segmentation, which extracts features and progressively restores details through an encoder‐decoder structure. The evaluation procedure is: (a) Both model predictions and ground‐truth annotations were converted into nucleotide‐level binary vectors, where “1” denotes gene regions and “0” denotes intergenic regions. (b) A position was considered predicted as “1” when the model output probability exceeded 0.5. (c) The Matthews Correlation Coefficient (MCC) was then calculated between the predicted and ground‐truth vectors. For the gene expression prediction task and the CREs strength prediction task, we added an MLP layer with 1 output as a regression head and assessed performance using PCC. For the TFBS prediction task and the chromatin accessibility prediction task, we adopted an MLP layer with 2 outputs as the classification head and employed both AUC and AUPRC as evaluation metrics.

### 5.7 Comparison with Other Gene Prediction Models

This evaluation used Helixer (version 0.3.4), ANNEVO (version 2.2), AUGUSTUS (version 3.3.3), and SegmentNT. Helixer employed a land plant‐specific model, ANNEVO used the embryophyte model, AUGUSTUS utilized a fine‐tuned model for a closely related species, and SegmentNT applied the multi‐species (SegmentNT‐MS) model. To ensure a fair comparison, the input sequence length for Helixer, ANNEVO, and AUGUSTUS was set to 65,536 bp. Considering that SegmentNT's maximum input length is limited to 50 kb, we used a sequence length of 49,152 bp for this tool.

### 5.8 Gene Generation Strategy

In gene sequence generation, a nucleus sampling strategy was adopted to generate candidate sequences ranging from 50 to 4000 bp in length. The temperature was set to 1.0 to preserve the original shape of the model's output probability distribution, while top‐p was set to 1.0 to allow sampling from the full probability distribution, maximizing expressive diversity. Additionally, top‐k was set to 24, restricting each step of sequence generation to the top 24 most probable candidates, thereby improving the overall quality and usability of the generated sequences. After generation, BLAST was used to align the candidate sequences against the 12 reference genomes from the pretraining phase to assess their novelty. The megablast mode was adopted with an e‐value threshold of 1e‐5 to filter out sequences with significant homology to the reference genomes, and max‐target‐seqs was set to 1 to retain only the best match for each candidate sequence.

### 5.9 Evo 2 Generation Strategy

We used the Evo 2–7B model for sequence generation, with Arabidopsis thaliana‐related prompts to guide biologically relevant outputs. Sampling parameters were set to temperature = 1, top‐p = 1, and top‐k = 4, with a maximum generation length of 4000 tokens, balancing diversity, stability, and sequence completeness.

### 5.10 MMD‐Based Feature Distribution Discrepancy

To measure the discrepancy between two feature distributions ***P*** and ***Q***, we used the Maximum Mean Discrepancy (MMD) Grettonetal.,2012 [50]. The empirical, unbiased, non‐squared MMD is computed as:

(5) $$MMD\;\left(X,Y\right)=\sqrt{\begin{array}{c} \frac{1}{n\left(n-1\right)}\sum_{i\neq j}k\left(x_{i},x_{j}\right)\\ +\frac{1}{m\left(m-1\right)}\sum_{i\neq j}k\left(y_{i},y_{j}\right)-\frac{2}{nm}\sum_{i,j}k\left(x_{i},y_{j}\right) \end{array}}\;$$

where $X=\{x_{i}\}\;\;∼\;P$, $Y=\{y_{i}\}\;\;∼\;Q$, and *k*(.,.) is the RBF kernel.

### 5.11 Vector construction

The vectors were constructed using a lighting cloning system (BDIT0014, Biodragon Immunotechnology). For gene transcription expression experiments, the synthesized full‐length (from 5’ UTR to 3’ UTR) gene sequences were cloned into the *EcoR1*/*Xba1*‐digested *pFGC5941*. For protein expression assays, the recombinant sequences, including the exons and introns of the gene as well as the GFP sequence fused at the C‐terminus, were cloned into the *EcoR1*/*Xba1*‐digested *pFGC5941*. The primer sequences are listed in Table S3.

### 5.12 Quantitative RT‐PCR Assays

Total RNA was isolated using Eastep Super Total RNA Extraction Kit according to manufacturer's protocol (LS1040, Promega). Reverse transcription reaction was performed using One‐Step gDNA Removal and cDNA Synthesis SuperMix for qPCR according to the manufacturer's protocol (AE311‐03, TransGen Biotech). The qPCR assays were performed on the LightCycler 480 II Detection System (Roche) using ChamQ Universal SYBR qPCR Master Mix (Q711‐02, Vazyme). The *NbEF1ɑ* gene was used as reference for gene expression in *N. benthamiana*. The relative expression level was calculated using the 2−ΔΔCT method. The primer sequences are listed in Table S4.

### 5.13 RNA‐Sequencing

RNA sequencing was performed on the DNBSEQ‐T7 platform (BGI Genomics Co., Ltd) using a 150 bp paired‐end sequencing strategy. A customized reference genome was constructed by integrating 7 synthetic exogenous gene sequences into the *Nicotiana benthamiana* genome (assembly NbeHZ1v1.0). The raw RNA‐seq reads were aligned to this modified reference genome using HISAT2 (version 2.1.0) with default parameters, followed by BAM file sorting with SAMtools. Transcriptome assembly and quantification were conducted using StringTie (version 2.1.4), with analysis restricted to uniquely mapped reads. For comprehensive data visualization, gene expression patterns were illustrated through heatmaps generated using the R package pheatmap. Visualize read alignment, genomic features, and their corresponding expression patterns within the genome using IGV (2.19.1). RNA sequencing datasets have been deposited to NCBI database with an accession number PRJNA1301568.

### 5.14 Protein Detection and Functional Assays

The recombinant constructs were individually transformed into Agrobacterium tumefaciens strain GV3101. The strains were resuspended in infiltration buffer (10 mm MES, pH 5.7, 10 mm MgCl2, and 150 µm acetosyringone) and incubated at 28°C for 2 h before infiltration into *N. benthamiana* leaf epidermal cells. Following a 48‐h incubation period, GFP fluorescence signals were detected utilizing a super‐resolution confocal scanning microscope (LSM980, Zeiss). Subsequently, the recombinant proteins were extracted employing RIPA lysis buffer (P0013B, Beyotime), and subjected to immunoblot analysis using a GFP antibody (MA5‐15256, Invitrogen).

### 5.15 Gene Expression Prediction

We employed a gene expression dataset derived from high‐quality transcriptomic data and corresponding genomic sequences from four plant species: Arabidopsis thaliana, rice, maize, and tomato. Each input sample was a 3,000 bp sequence composed of 2,000 bp upstream of the transcription start site (TSS), 300 bp downstream of the TSS, 500 bp upstream of the transcription termination site (TTS), and 200 bp downstream of the TTS. The gene expression value associated with each sequence was used as the regression target. To evaluate the model's generalization ability, we designated specific chromosomes from each species as held‐out test sets with no overlap with the training data: chromosome 5 for Arabidopsis, chromosomes 7 and 8 for rice, chromosomes 5 and 6 for maize, and chromosomes 7, 8, and 9 for tomato. We followed the benchmark setup provided by PlantCRE (https://github.com/liulifenyf/PlantCRE), with detailed sample sizes listed in Table S7.

### 5.16 Chromatin Accessibility Prediction

We employed chromatin accessibility dataset derived from ATAC‐seq data which encompasses multiple tissues from six plant species: Arabidopsis thaliana, rice, maize, millet, sorghum, and Brachypodium distachyon [40]. For each ATAC‐seq sample, we labeled each training sequence as 1 (positive sample) if the central 200 bp region overlapped with an OCR by more than 50% of its length; otherwise, it was labeled as 0 (negative sample), with each sample being 1000 bp in length. We chose maize, a crop of significant global economic value, as a representative to assess our model. To ensure a fair comparison with the model developed by Zhao et al., we sourced our training and testing datasets from https://plantdeepsea‐toturial2.readthedocs.io/en/latest/08‐Statistics.html#training‐data, which comprised 6,479,872 training samples and 79,872 testing samples.

### 5.17 TFBS Prediction

We utilized a recently developed TFBS dataset, which includes 385 TFs from three species: Arabidopsis, rice, and maize. We selected transcription factors for which the TSPTFBS2.0 model could not achieve good predictive results (ACC less than 0.95) as our evaluation dataset, including 96 maize TFs, 7 rice TFs, and 1 Arabidopsis TF [57]. The input sequences in this dataset were set to a length of 500 bp, extending 250 bp to both the left and right of the peak regions. To ensure a fair comparison, we obtained the transcription factor binding site data from https://github.com/liulifenyf/TSPTFBS‐2.0/tree/main/Data.

### 5.18 CREs Strength Prediction

We employed two datasets: the core promoter and the terminator dataset. The core promoter dataset consists of 18,329 Arabidopsis, 34,415 maize, and 27,094 sorghum core promoters, each 170 bp in length, ranging from −165 to +5 bp relative to the annotated Transcription Start Site (TSS), tested for activity strength in two systems through STARR‐seq experiments. The terminator dataset consists of 24,529 Arabidopsis and 30,092 maize terminators, each 170‐bp in length, ranging from −150 to +20 bp relative to the Cleavage and Polyadenylation Site (CPS), tested for activity strength in maize protoplast and tobacco leaf systems under long‐day conditions (16 h of light and 8 h of dark) through STARR‐seq. To ensure a fair comparison with existing models, we downloaded the training and testing datasets for plant promoters and terminators from http://www.hzau‐hulab.com/icrepcp/download/ and https://github.com/lampoona/Terminators‐Plant‐STARR‐seq, respectively. The number of Fine‐tuning and testing samples for core promoters and terminators can be found in Table S8. These datasets enabled the standardized evaluation of different predictive models. Extended Data Figure 2 presents a comparison of the performance of various models in predicting the strength of plant CREs.

### 5.19 Comparison with Other Genomic Language Models

We systematically compared PlantGFM with several representative large genome models, including AgroNT, NT‐v2‐250 m, NT‐v2‐100 m, and DNABERT2, across multiple tasks such as gene expression prediction, transcription factor binding site prediction, chromatin open region prediction, and plant promoter and terminator prediction. For fine‐tuning, we applied full‐parameter fine‐tuning to NT‐v2‐250 m, NT‐v2‐100 m, and DNABERT2 across all tasks, while for AgroNT, we employed parameter‐efficient fine‐tuning based on IA3 (Infused Adapter by Inhibiting and Amplifying Inner Activations) [64]. All models were optimized under multiple hyperparameter settings, and the best‐performing results on the validation set were selected for final reporting. Detailed information is provided in Table S11.

### 5.20 Hardware and Computational Resources

All pre‐training, fine‐tuning, and retraining processes were conducted using the Torch framework and the Hugging Face Transformers library. The pre‐training of PlantGFM was performed on 8 Nvidia A800‐80G GPUs for a total of 468 h, divided into three stages: 2, 36, and 430 h respectively, until convergence was achieved. The fine‐tuning for the gene prediction task was also carried out on 8 Nvidia A800‐80G GPUs, reaching convergence within 42 h. Other fine‐tuning or generation tasks were completed using 2 to 8 Nvidia RTX 4090 GPUs, depending on the scale of the dataset.

## Author contributions

X.‐H.H., Q.‐Y.Y. and J.‐B.Y. designed the research and wrote the manuscript. C.‐H.L., Q.‐Z.Z. and K.‐P.L. collected the data and built the PlantGFM model. Q.‐Z.Z. performed the gene prediction task. C.‐H.L. performed the sequence generation task. H.‐C.C. performed the experimental validations on 7 generating sequences and C.‐F.L. analyzed the experimental data. C.‐H.L. and Q.‐Z.Z. performed four regulatory genomics tasks. All authors read and approved the final manuscript.

## Funding

This research was supported by the National Key Research and Development Program of China (2023YFD1202903 to X.‐H.H.) and the National Natural Science Foundation of China (32322061 and 32441059 to Q.‐Y.Y.).

## Conflicts of Interest

The authors declare no conflicts of interest.

## Supporting information




**Supporting File 1**: advs75772‐sup‐0001‐SuppMat.docx.

**Supporting File 2**: advs75772‐sup‐0002‐TableS3.xlsx.

**Supporting File 3**: advs75772‐sup‐0003‐TableS5.xlsx.

**Supporting File 4**: advs75772‐sup‐0004‐TableS6.xlsx.

**Supporting File 5**: advs75772‐sup‐0005‐TableS11.xlsx.

## Data Availability

All the datasets that used to build the PlantGFM models and all the trained PlantGFM models are available at https://huggingface.co/hu‐lab. RNA sequencing datasets have been deposited to NCBI database with an accession number PRJNA1301568.
