# Supplementary material for: PlantGFM: A Genomic Foundation Model for Discovery and Creation of Plant Genes
Source: Adv Sci (Weinh). 2026 May 20:e75772. Online ahead of print. doi: 10.1002/advs.75772 (PMC13336098; doi:10.1002/advs.75772)
Supplement: Supplementary file 1 — Supporting File 1: advs75772‐sup‐0001‐SuppMat.docx. [file ADVS-9999-e75772-s004.docx]

**Supporting information**

**Figure S1.** Performance comparison of gene prediction models for exon and coding CDS prediction.

**Figure S2.** Comparison of gene prediction performance across models with different tokenization strategies and context lengths.

**Figure S3.** Comparison of sequence features between natural genes and model-generated sequences.

**Figure S4.** Comparative Analysis of Conserved Splice Site Motifs in Natural and Generated Sequences. **Figure S5.** Comparison of Helixer and Augustus annotations for 7 *de novo*–AI-generated sequences.

**Figure S6.** MMD comparison of embedding distributions for natural, generated, and random gene sequences.

**Figure S7.** Independent biological replicates of immunoblot analysis shown in Figure 4E.

**Figure S8.** Overlap of DEGs between S631 and S720.

**Table S1.** Detailed information on the species names, abbreviations, genome versions, and data sources included in the pretraining corpus.

**Table S2.** Species and Chromosome Selection for Gene Prediction Validation

**Table S3.** Sequence information of AI-generated sequences. Note: Due to its large size, Table S3 is provided as a separate Excel file.

**Table S4.** Primer sequences used in this study.

**Table S5.** List of differentially expressed genes (DEGs) in tobacco leaves overexpressing S631 relative to the empty-vector control. Note: Due to its large size, Table S5 is provided as a separate Excel file.

**Table S6.** List of differentially expressed genes (DEGs) in tobacco leaves overexpressing S720 relative to the empty-vector control. Note: Due to its large size, Table S6 is provided as a separate Excel file.

**Table S7.** Fine-tuning and Testing of Four Species for gene expression prediction task.

**Table S8.** Fine-tuning and Testing Sample Numbers in Various Transient Transfection Systems

**Table S9.** Composition of the tokenizer vocabulary used in PlantGFM.

**Table S10.** Detailed hyperparameters and training settings for the three-stage pre-training process.

**Table S11.** Detailed hyperparameters for baseline CNN and genomic language models. Due to its large size, Table S11 is provided as a separate Excel file.

**
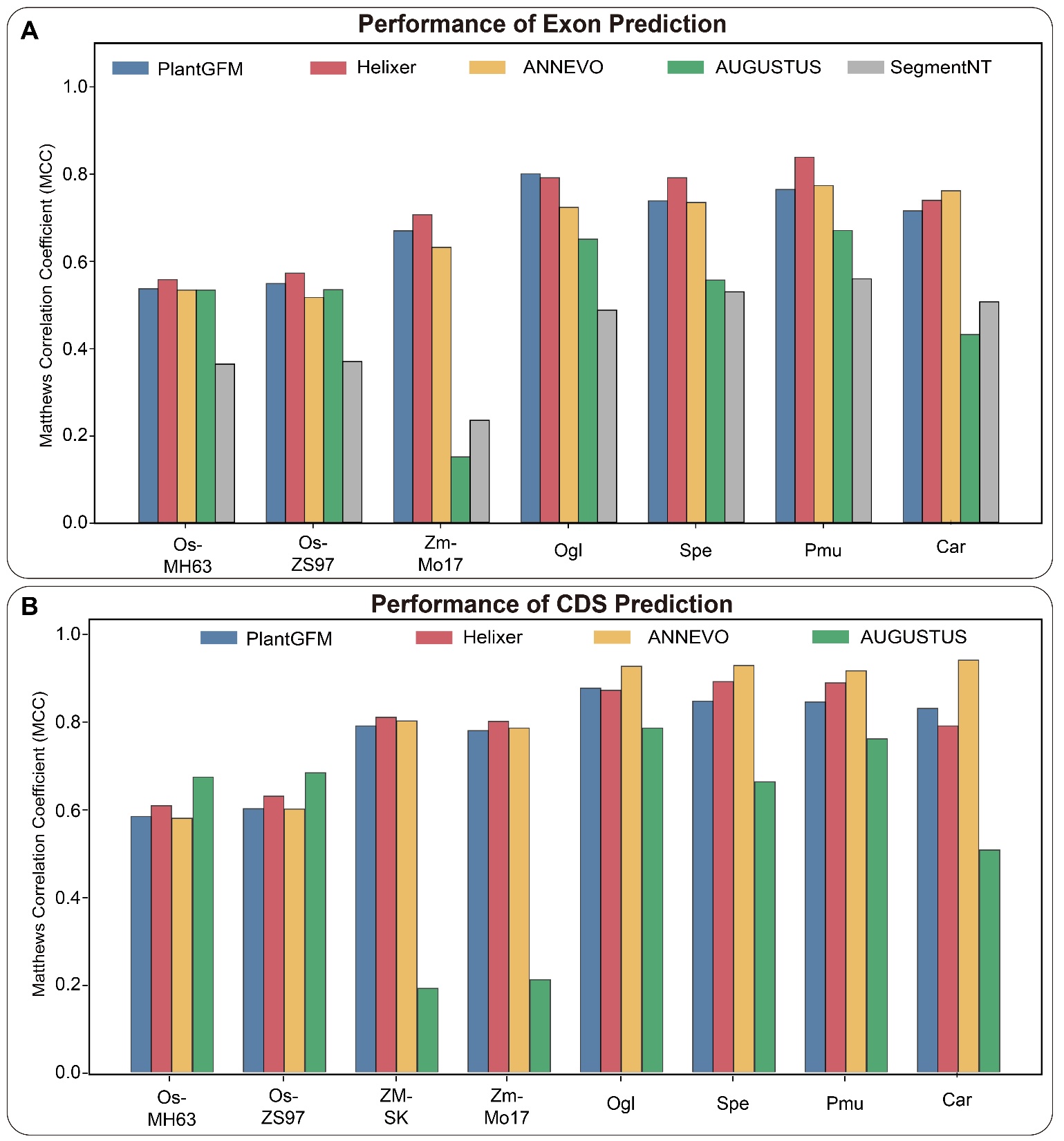
**

**Figure S1.** Performance comparison of gene prediction models for exon and coding CDS prediction. **A)** Exon prediction model performance. The model, using the same architecture as PlantGFM, was fine-tuned on exon annotations from eight species (acv, ath, bra, bni, osa, ppa, sly, smo; vca, bol, and gma lacked exon annotations in this version). Model performance was compared against Helixer, ANNECO, Augustus, and SegmentNT. **B)** Coding sequence (CDS) prediction model performance. The model, using the same architecture as PlantGFM, was fine-tuned on CDS annotations from twelve species. Model performance was compared against Helixer, ANNEVO, Augustus, and SegmentNT.


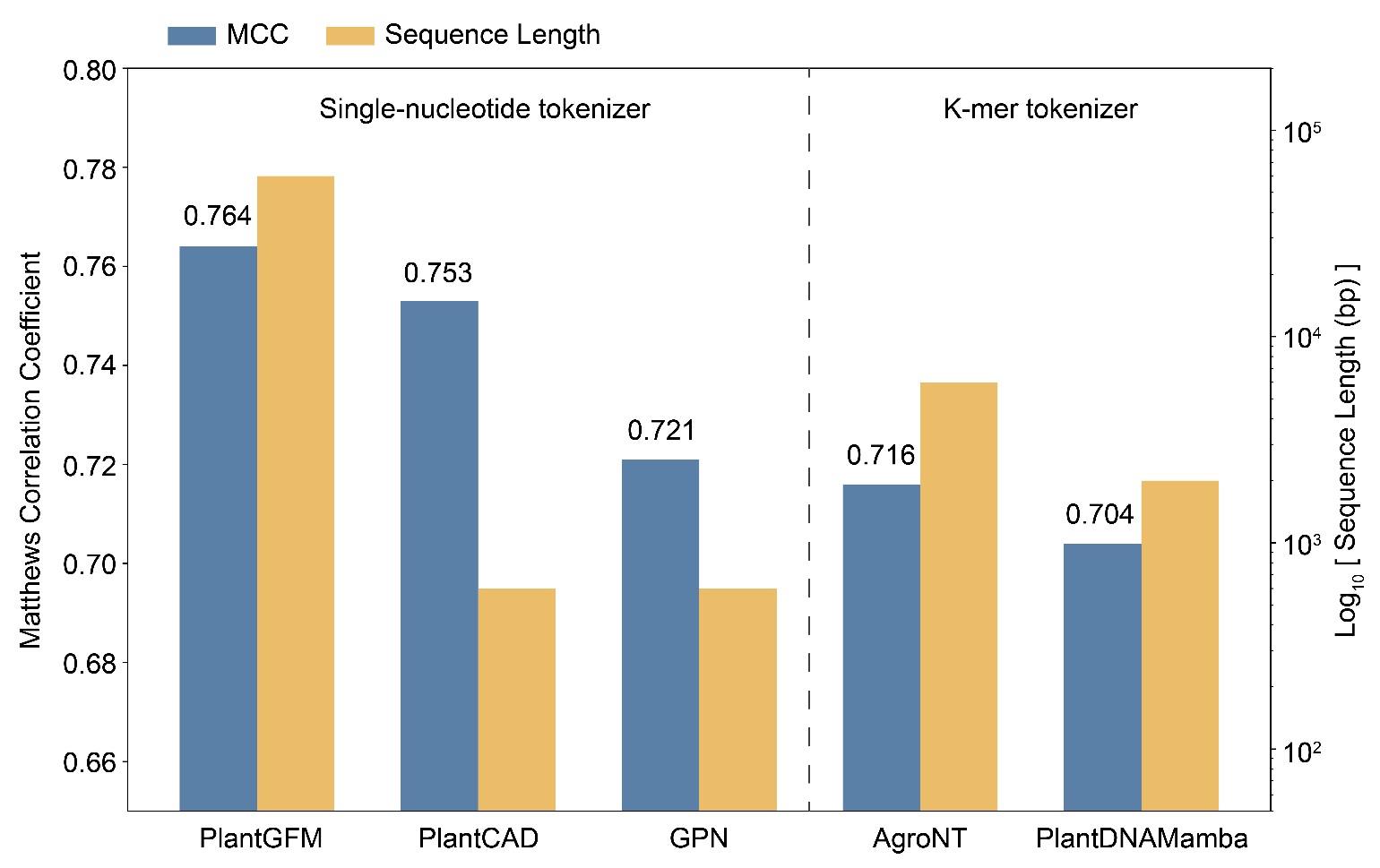


**Figure S2.** Comparison of gene prediction performance across models with different tokenization strategies and context lengths.


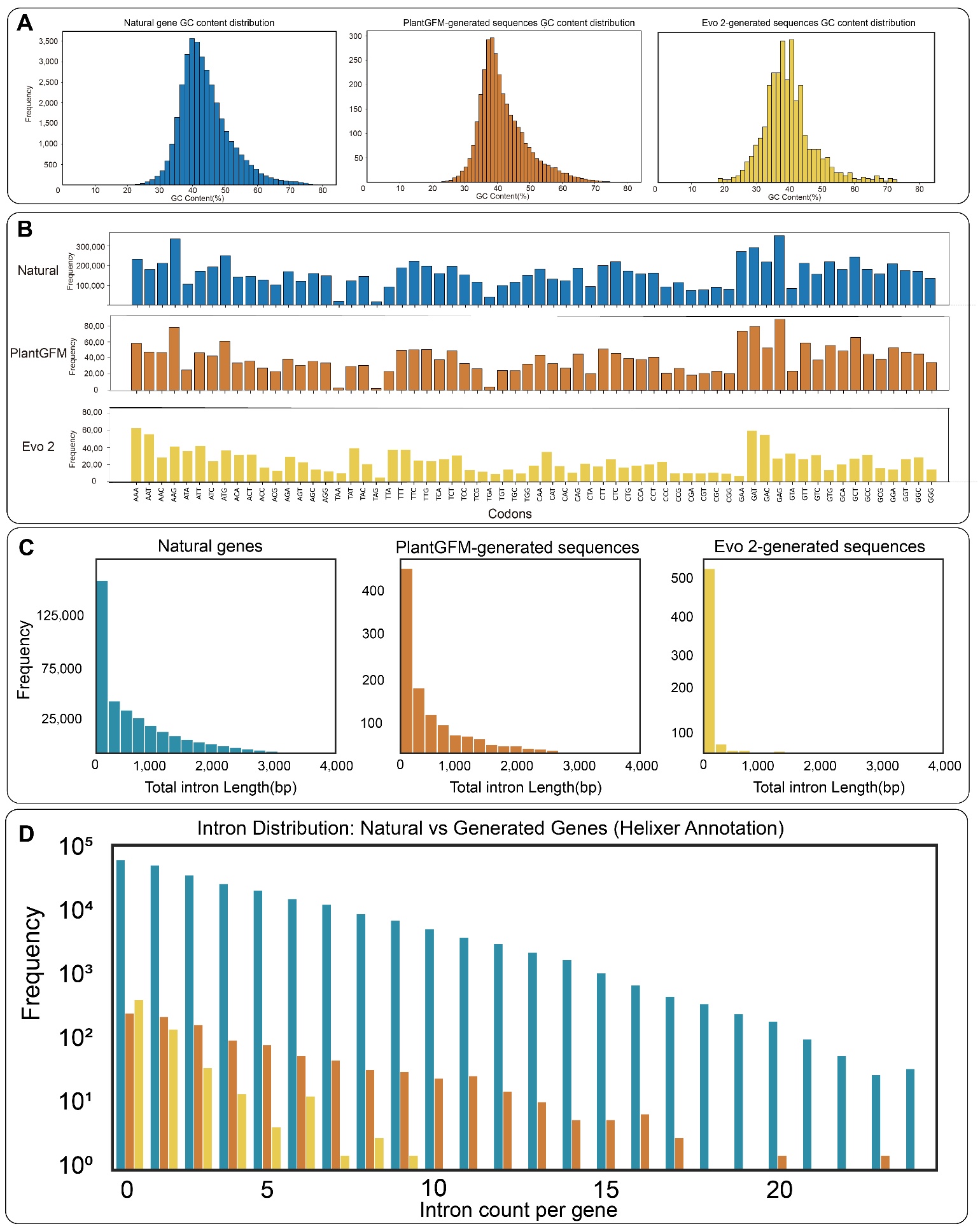


**Figure S3.** Comparison of sequence features between natural genes and model-generated sequences. **A)**. GC content of natural genes and sequences generated by PlantGFM and Evo 2 (based on Helixer annotations) are shown. **B).** Codon usage frequencies across all 64 codons are compared. PlantGFM better reproduces the specific codon preferences of natural sequences, whereas Evo 2 shows a more generalized distribution. **C).** Sequences generated from Helixer annotations show that PlantGFM better reproduces long introns and the overall intron length distribution than Evo 2, more closely reflecting natural gene architecture. **D).** Sequences generated from Helixer annotations show that PlantGFM captures more complex intron structures than Evo 2, better reflecting natural gene architecture.


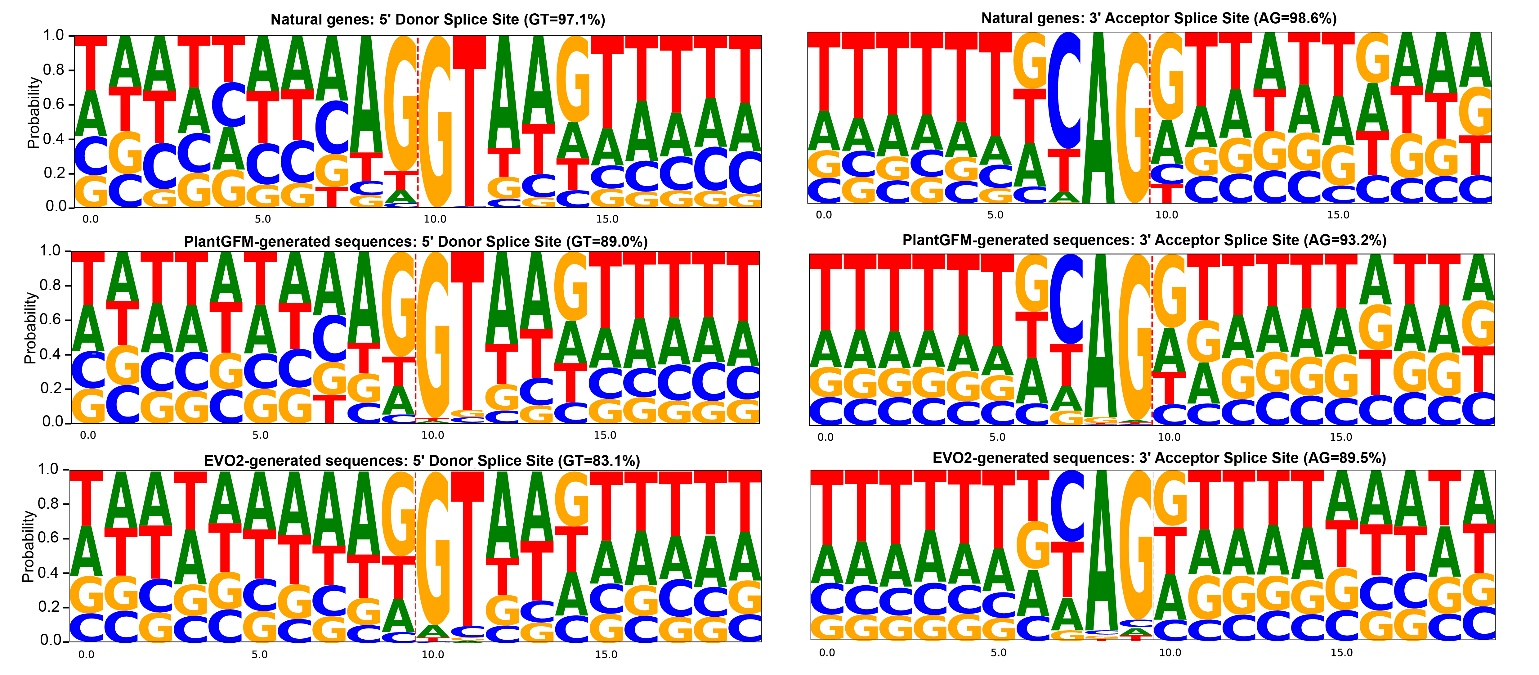


**Figure S4.** Comparative Analysis of Conserved Splice Site Motifs in Natural and Generated Sequences. Based on Helixer annotations, PlantGFM more accurately reproduces conserved 5′ donor (GT) and 3′ acceptor (AG) splice site motifs than Evo 2, closely matching the patterns seen in natural genes.


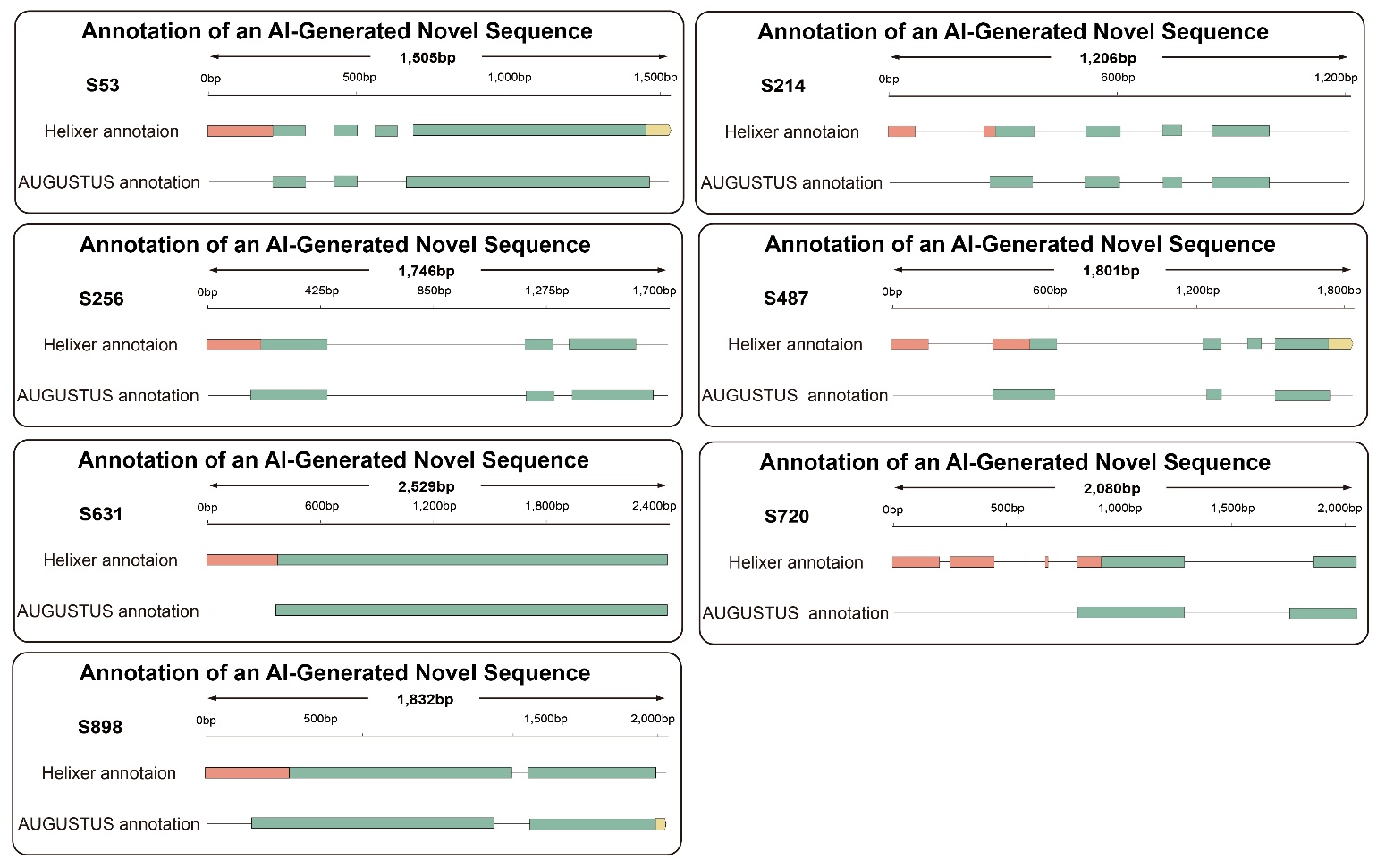


**Figure S5.** Comparison of Helixer and Augustus annotations for 7 *de novo*–AI-generated sequences. Gene IDs and total gene lengths are shown at the top of each panel, which displays the gene structure predictions by Helixer (middle) and AUGUSTUS (bottom), with coding sequences, 5′ UTR, and 3′ UTR indicated.


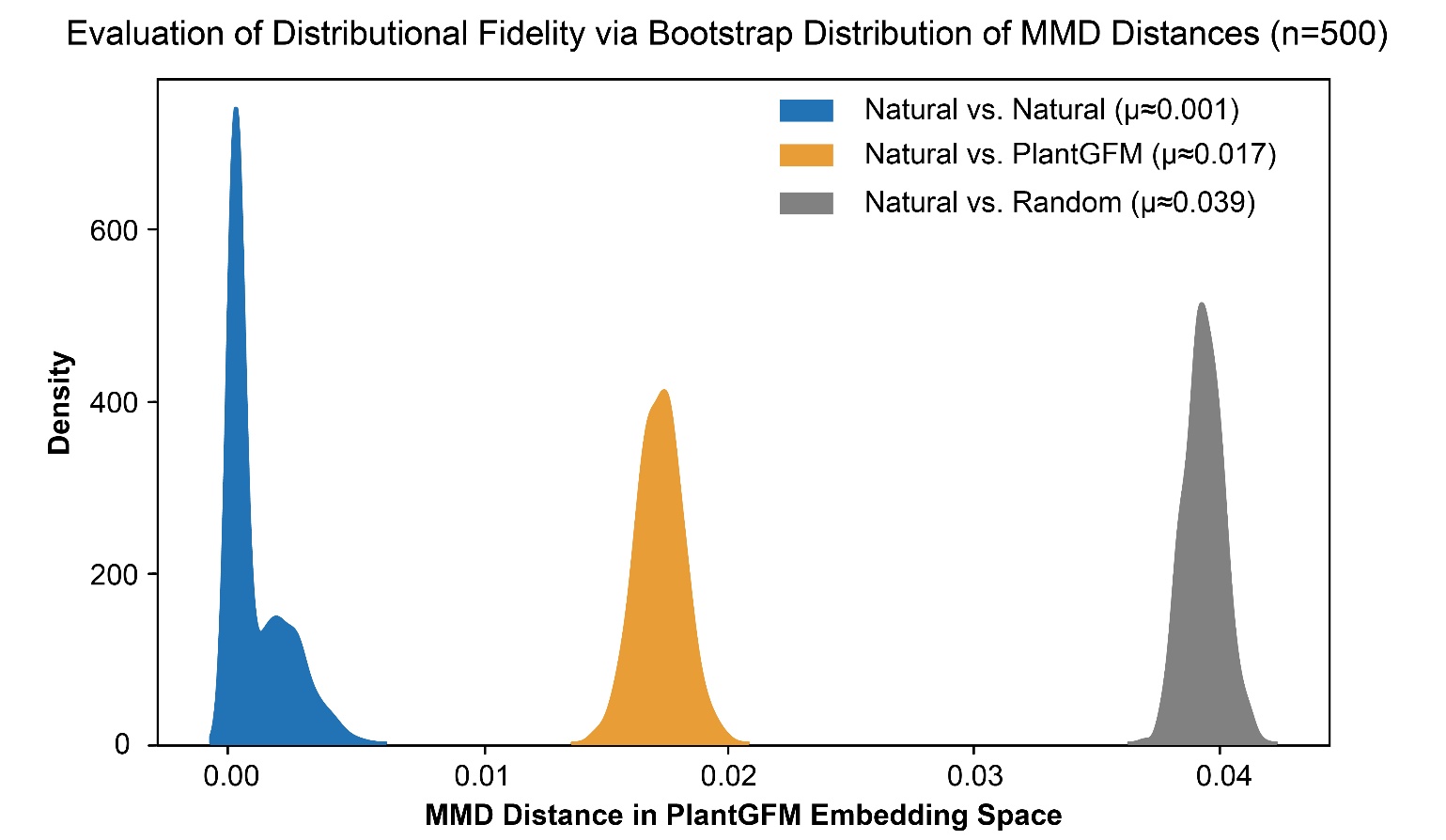
**Figure S6.** Evaluation of distributional fidelity using PlantGFM embeddings. Distribution of MMD distances ($\sqrt{MMD^{2}}$) was calculated based on high-dimensional features extracted from the PlantGFM embedding space. To ensure a fair comparison despite the large population of natural sequences (N = 355，910), a bootstrap resampling approach (n = 500 iterations) was employed, drawing samples per trial. Natural vs. Natural serves as the baseline for internal distribution variance, while Natural vs. PlantGFM demonstrates the generative fidelity of our model by closely approaching this baseline. Both distributions outperform the Natural vs. Random negative control.


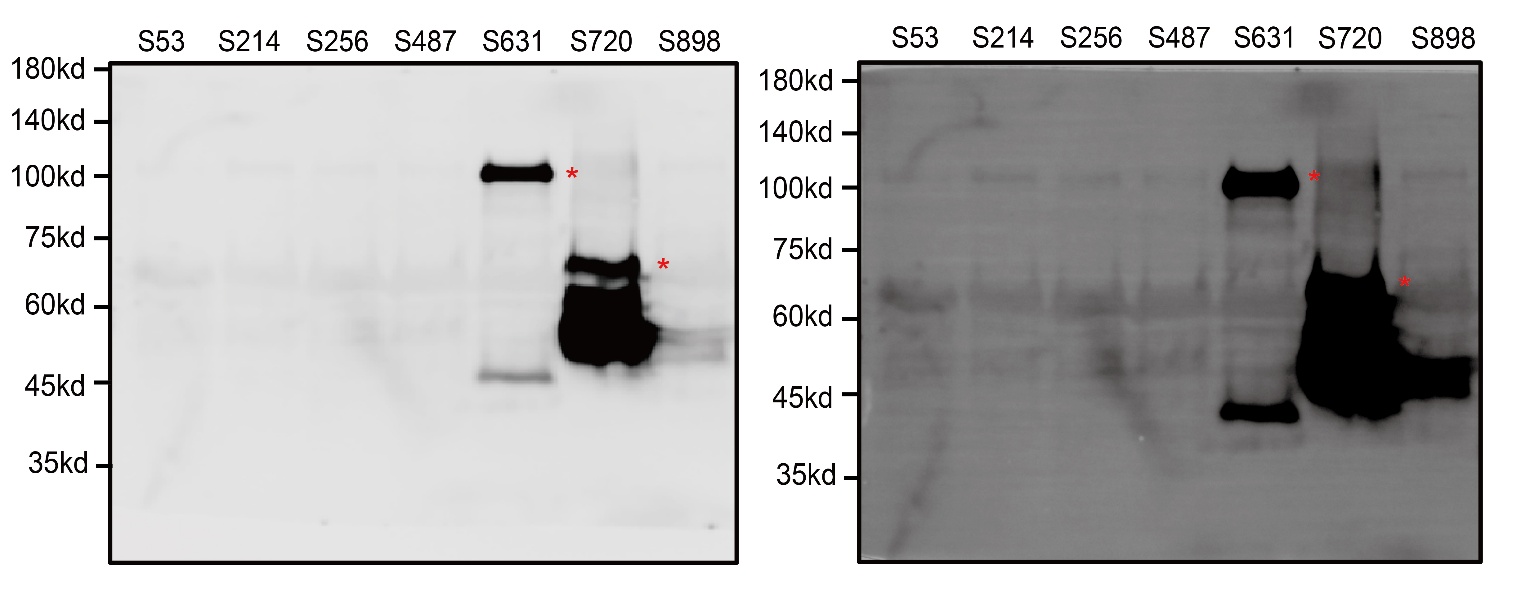


**Figure S7.** Independent biological replicates of immunoblot analysis shown in Figure 4E.


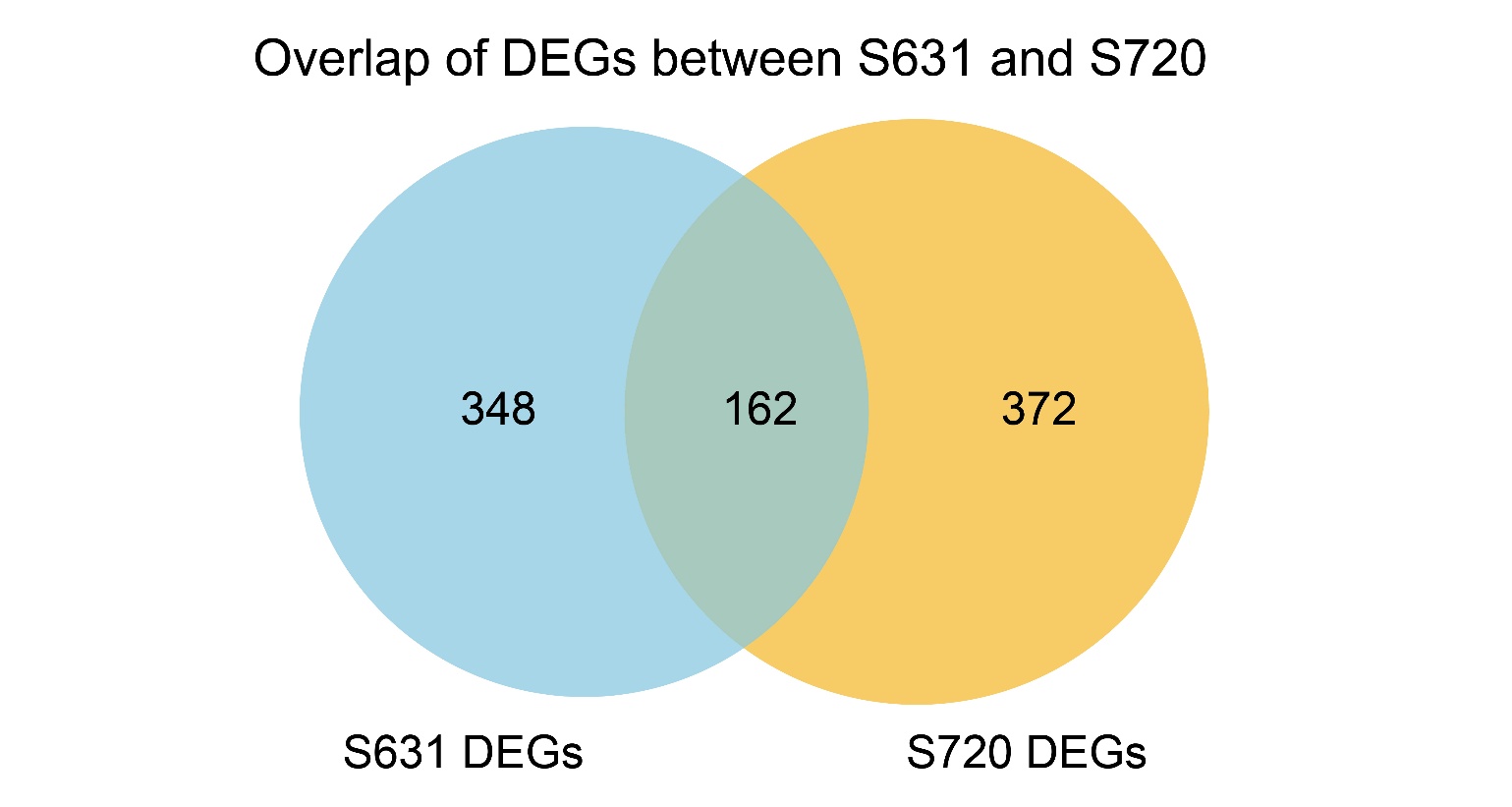


**Figure S8.** Overlap of DEGs between S631 and S720.

**Table S1.** Detailed information on the species names, abbreviations, genome versions, and data sources included in the pretraining corpus.****

**Table S2.** Species and Chromosome Selection for Gene Prediction Validation

**Table S4.** Primer sequences used in this study

**Table S7.** Fine-tuning and Testing of Four Species for gene expression prediction task

**Table S8.** Fine-tuning and Testing Sample Numbers in Various Transient Transfection Systems

**Table S9.** Composition of the tokenizer vocabulary used in PlantGFM.

**Table S10.** Detailed hyperparameters and training settings for the three-stage pre-training process.
